# Apoptotic exosome-like vesicles transfer specific and functional mRNAs to endothelial cells by phosphatidylserine-dependent macropinocytosis

**DOI:** 10.1038/s41419-023-05991-x

**Published:** 2023-07-20

**Authors:** Alexandre Brodeur, Francis Migneault, Maude Lanoie, Déborah Beillevaire, Julie Turgeon, Annie Karakeussian-Rimbaud, Nicolas Thibodeau, Éric Boilard, Mélanie Dieudé, Marie-Josée Hébert

**Affiliations:** 1 Centre de Recherche, Centre Hospitalier de l’Université de Montréal (CRCHUM) and Université de Montréal, Montréal, QC Canada; 2 Canadian Donation and Transplantation Research Program (CDTRP), Edmonton, AL Canada; 3 Département de Médecine, Université de Montréal, Montréal, QC Canada; 4 Centre de Recherche, Centre Hospitalier Universitaire (CHU) de Québec-Université Laval, Département de Microbiologie et Immunologie, Québec, QC Canada; 5 Département de Microbiologie, Infectiologie et Immunologie, Faculté de Médecine, Université de Montréal, Montréal, QC Canada; 6 Héma-Québec, Québec, QC Canada

**Keywords:** Small molecules, RNA

## Abstract

Apoptosis of endothelial cells prompts the release of apoptotic exosome-like vesicles (ApoExos), subtype extracellular vesicles secreted by apoptotic cells after caspase-3 activation. ApoExos are different from both apoptotic bodies and classical exosomes in their protein and nucleic acid contents and functions. In contrast to classical apoptotic bodies, ApoExos induce immunogenic responses that can be maladaptive when not tightly regulated. In the present study, we elucidated the mechanisms by which ApoExos are internalized by endothelial cells, which leads to shared specific and functional mRNAs of importance to endothelial function. Using flow cytometry and confocal microscopy, we revealed that ApoExos were actively internalized by endothelial cells. SiRNA-induced inhibition of classical endocytosis pathways with pharmacological inhibitors showed that ApoExos were internalized via phosphatidylserine-dependent macropinocytosis independently of classical endocytosis pathways. An electron microscopy analysis revealed that ApoExos increased the macropinocytosis rate in endothelial cells, setting in motion a positive feedback loop that increased the amount of internalized ApoExos. Deep sequencing of total RNA revealed that ApoExos possessed a unique protein-coding RNA profile, with PCSK5 being the most abundant mRNA. Internalization of ApoExos by cells led to the transfer of this RNA content from the ApoExos to cells. Specifically, PCSK5 mRNA was transferred to cells that had taken up ApoExos, and these cells subsequently expressed PCSK5. Collectively, our findings suggest that macropinocytosis is an effective entry pathway for the delivery of RNAs carried by ApoExos and that these RNAs are functionally expressed by the endothelial cells that internalize them. As ApoExos express a specific mRNA signature, these results suggest new avenues to understand how ApoExos produced at sites of vascular injury impact vascular function.

## Introduction

Intercellular communication involves a set of mechanisms that allow a cell, a tissue, and an organ to receive, interpret, and respond to signals emitted from other cells, regardless of whether these cells are in the same environment or distally located. Signals can be mediated through direct contact between cells or through the transfer of secreted molecules. In the past decade, the intercellular transfer of extracellular vesicles (EVs) has emerged as a novel and important mechanism of intercellular communication. EVs are now recognized as key players in shaping phenotypic changes in the cells that internalize them and in organ-to-organ communication [1, 2].

Extracellular vesicles are composed of a lipid bilayer membrane enclosing bioactive molecules, including RNAs and proteins. EVs are categorized into different groups depending on their biogenesis, size, and biomarkers [3, 4]. Microvesicles and apoptotic bodies (ApoBodies) are generated by direct shedding of the plasma membrane, and their size ranges from 100 nm to a few microns. On the other hand, exosomes are small (30–100 nm) membranous vesicles generated within the endosomal system [5] from inward budding of the limiting membrane of multivesicular bodies (MVBs) followed by fusion of MVBs with the plasma membrane and release of the MVBs (EVs) into the extracellular environment [6, 7]. Exosomes can bind to the cell surface to trigger intrinsic signaling pathways [8] or can be internalized by recipient cells through membrane fusion or endocytic mechanisms [9]. Various mechanisms of exosome uptake have been described, with clathrin-mediated and caveolae-mediated endocytosis emerging as key uptake mechanisms [2]. Other means of entry have also been described, such as lipid raft-mediated endocytosis [10], specific protein‒protein interaction-induced internalization [11], and macropinocytosis [12].

Our group described apoptotic exosome-like vesicles (ApoExos) as a subtype of extracellular vesicles with sizes similar to those of exosomes but that are produced through different biogenesis mechanisms and that carry different markers. Within the exosome size range, ApoExos express protein biomarkers, such as the autoantigen perlecan/LG3 and active 20S proteasome core complex, which are not carried by classical exosomes (ExoN) or apoptotic bodies [13–16]. Although they express certain exosome markers, such as syntenin and TCTP, ApoExos lack classic exosome markers, such as tetraspanins (CD9, CD63, and CD81). In contrast to classical exosomes that are generated from late endosomes through inward budding of the multivesicular body membrane, ApoExos are formed in stressed cells within large autolysosomes and released into the extracellular environment after caspase-3 activation. Caspase-3 plays an active role in ApoExos release in the extracellular environment by driving fusion between autolysosomes and the cell membrane [16]. Thus, ApoExos biogenesis differs from that of apoptotic bodies, which stem from cell membrane blebbing, and ApoExos lack characteristic markers of apoptotic bodies such as HMGB1. In contrast to apoptotic bodies, known for their noninflammatory and tolerogenic properties, ApoExos foster robust proinflammatory and autoimmune responses [13, 15, 17]. They drive NF-κB activation in endothelial cells, inducing endothelial cell dysfunction and dedifferentiation [18]. ApoExos also differ from both classical exosomes and apoptotic bodies on the basis of their RNA and protein content and enzymatic activity. When ApoExos RNA content was compared to that of classical exosomes or apoptotic bodies, ApoExos were found to harbor unique immunostimulatory virus-like RNAs [19]. Whether ApoExos can carry unique cargo, such as RNAs that are not carried by either classical exosomes or apoptotic bodies, remains to be evaluated. In the present study, we aimed to elucidate the mechanisms of ApoExo uptake by endothelial cells and determine whether this uptake leads to specific and functional mRNAs important to endothelial function being shared with endothelial cells.

## Results

### Apoptotic exosome-like vesicles are actively internalized by endothelial cells

**Fig. 1:**
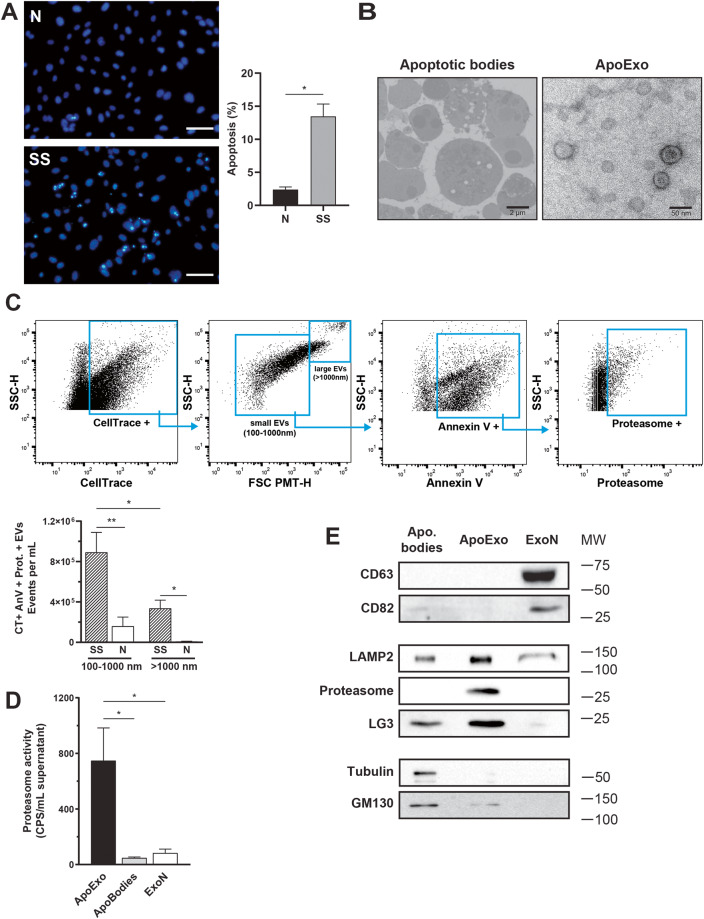
Apoptotic exosome-like vesicles (ApoExos) are different from classical exosomes (ExoNs) and apoptotic bodies (ApoBodies). **A** Quantification of apoptosis levels in endothelial cells exposed to normal medium (N) or serum-starved medium (SS) for 4 h in representative micrographs. Fluorescence microscopy of endothelial cells stained with Hoechst and propidium iodine. Apoptotic and total cell numbers were quantified using ImageJ software. Apoptosis levels are expressed as the mean ± SEM. *n* ≥ 3 for each condition. Scale bar: 200 µm. **B** Representative transmission electron micrographs of ApoBodies (left, scale bar: 2 µm) and ApoExos (right, scale bar: 50 and 100 nm). *n* ≥ 3 under each condition. **C** Gating strategy to analyze the expression of phosphatidylserine and proteasome on CellTrace + EVs in conditioned medium from endothelial cells and representative dot plot based on the supernatant of serum-starved endothelial cells. Flow cytometric quantifications of CellTrace (CT) + Annexin V (AnV) + Proteasome (Prot) + extracellular vesicles detected in the supernatant of endothelial cells; *n* ≥ 5 for each condition. **D** Comparison of proteasome caspase-like activity in ApoExos, ApoBodies and classical exosomes (ExoN). Values are expressed as the mean ± SEM. *n* ≥ 4 for each condition. **E** Western blots showing various protein markers in ApoBodies, ApoExo, and ExoN. Molecular weights are expressed in kDa. Representative blots are depicted. *n* ≥ 3 for each condition. *P* values were obtained by unpaired t test (*P < 0.05, **P < 0.01, and ***P < 0.001).

Endothelial cells exposed to serum starvation activate a proapoptotic response without necrotic features (Fig. 1A). Using electron microscopy and high-sensitivity flow cytometry (hs-FACS), we confirmed that endothelial cells under normal or serum-free proapoptotic conditions released extracellular vesicles of different sizes. Apoptotic bodies and ApoExos isolated by sequential centrifugation from serum-free medium used to culture apoptotic endothelial cells presented distinct ultrastructural characteristics, with apoptotic bodies in the micron size range and ApoExos between 30 and 100 nm in diameter (Fig. 1B). Using an LWA300 proteasome probe, we detected and quantified active proteasome-containing extracellular vesicles directly in medium used to culture endothelial cells [20]. Supernatant from apoptotic serum-starved endothelial cells but not normal controls contained phosphatidylserine-positive vesicles in the 100–1000 nm size range that harbored active proteasome activity characteristic of ApoExos (Fig. 1C). The detection specificity of extracellular vesicles by hs-FCM was confirmed by using a combination of validation experiments. Specifically, we confirmed the particle size, nature, and membrane moiety of extracellular vesicles by using silica particles, ultracentrifugation depletion, Ca^2+^ ion chelation, and detergent treatment, respectively (Fig. S1). Consistent with hs-FAC data, proteasome caspase-like activity was enriched in the ApoExo fraction compared to apoptotic body and classical exosome fractions obtained from healthy endothelial cells (Fig. 1D). An immunoblot analysis confirmed that ApoExos expressed distinct markers, such as LAMP2, LG3, and the 20S proteasome, in contrast to the markers carried by apoptotic bodies, which include GM130 and tubulin. ApoExos lack classical tetraspanin markers such as CD63 and CD82, which are expressed in exosomes produced by normal “nondying” cells (Fig. 1E).

**Fig. 2:**
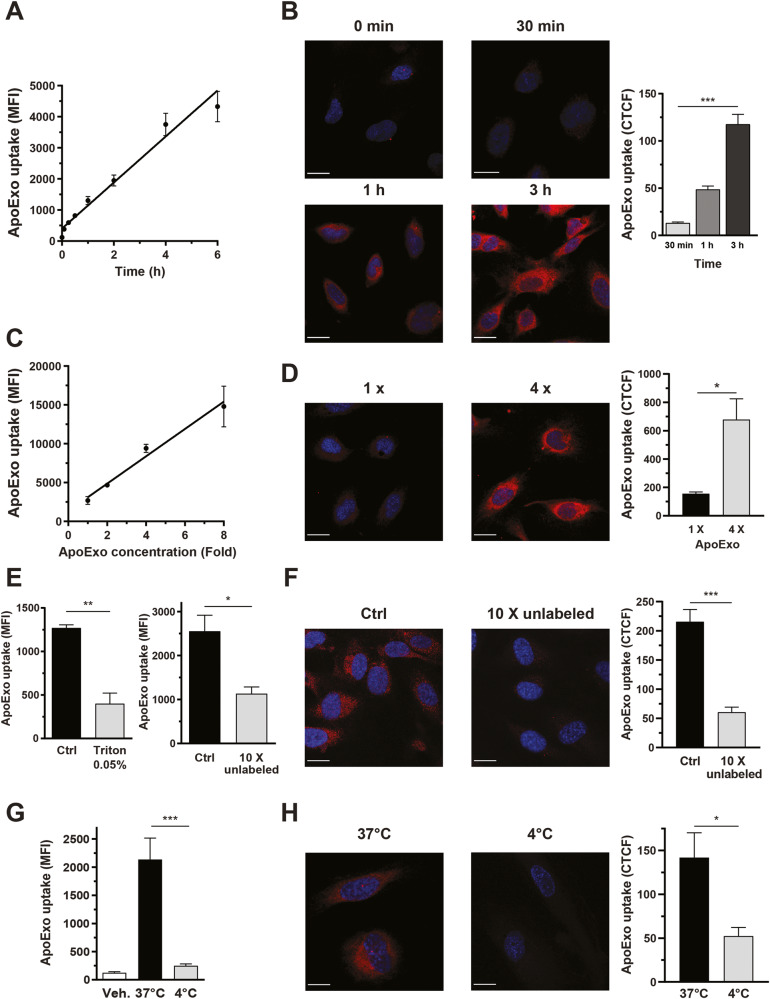
Apoptotic exosome-like vesicles are actively internalized by an energy-dependent mechanism. Time-dependent uptake of protein-labeled ApoExo by endothelial cells under serum starvation from 1 to 6 h as quantified by flow cytometry (**A**) or confocal microscopy (**B**). Concentration-dependent uptake of protein-labeled ApoExos by serum-starved endothelial cells for more than 1 h as quantified by flow cytometry (**C**) or confocal microscopy (**D**). **E** Quantification by flow cytometry data showing ApoExo uptake by serum-starved endothelial cells incubated for 1 h with ApoExos treated with 0.05% Triton X-100 (left) or an excess (10X) of unlabeled vesicles. **F** Quantification of ApoExos uptake by serum-starved endothelial cells incubated for 1 h with ApoExo treated with an excess (10X) unlabeled vesicles, by confocal microscopy. Quantification of ApoExo uptake by serum-starved endothelial cells treated at 37 °C or 4 °C for 1 h using flow cytometry (**G**) or confocal microscopy (**H**). Flow cytometry experiments are expressed as the median fluorescence intensity (MFI) or cell fluorescence percentage of vehicle-treated cells MFI (30,000 events/sample) ± SEM. Confocal microscopy experimental data are expressed as corrected total cell fluorescence (CTCF) ± SEM. Representative pictures of ApoExo internalization taken with a confocal microscope (scale bar: 20 µm; red—ApoExo and blue—nucleus). Control (Ctrl) represents cells treated with protein-labeled ApoExos after serum starvation. Vehicle (Veh.) represents cells treated with RPMI serum-free medium. ApoExo basal concentration (1 X) is based on the initial number of parental cells and used at the same concentration for the experimental procedures on recipient cells. *n* ≥ 3 for each condition. *P* values were obtained by unpaired t test (*P < 0.05, **P < 0.01, and ***P < 0.001).

We then investigated whether endothelial cells internalized ApoExos with the intent of identifying mechanisms that control this internalization. Protein-labeled ApoExos were generated from serum-starved endothelial cells treated with a fluorescent dye that becomes cell-impermeant after covalently binding of glutathione-*S*-transferase to proteins. This fluorescent dye consisted of a chloromethyl group that reacts with thiol groups of proteins. Therefore, all proteins containing thiol groups loaded into ApoExos were labeled. This dye remains stable in living and daughter cells and EVs [21, 22]. Endothelial monolayers were exposed to fluorescently labeled ApoExos for as long as 6 h, and internalization was studied by flow cytometry and confocal microscopy. The efficient uptake of ApoExos by endothelial cells was observed in a time- and concentration-dependent manner, and confocal microscopy confirmed that internalized ApoExos localized to the cytoplasm not the cell surface (Fig. 2A–D). The specificity of ApoExo uptake was supported by a significant decrease in the fluorescence signal following concomitant exposure of the cells to unlabeled ApoExos (Fig. 2E, F). Treatment of fluorescently labeled ApoExos with Triton X-100 prevented their uptake by endothelial cells (Fig. 2E), confirming that uptake was dependent on the presence of intact membrane vesicles. We then evaluated whether the uptake of ApoExos depended on an active process by incubating endothelial cells with ApoExos at 4 °C. The ApoExo uptake rate, as well as the uptake rate of ExoNs and ApoBodies, was significantly decreased at 4 °C (Fig. 2G, H, Fig. S2A), suggesting that metabolic activity in endothelial cells was necessary for the uptake of extracellular vesicles. However, lowering the temperature to 4 °C changed the viscosity of the cell medium, which could also affect the uptake rate. Internalization of ApoExos was not restricted to HUVECs, as murine primary renal endothelial cells and WI38 human fibroblasts similarly internalized ApoExos (Fig. S2B). Collectively, these results suggest that cells actively internalize ApoExos through an energy-dependent mechanism.

### Apoptotic exosome-like vesicles are internalized via a nonclassical endocytosis pathway

**Fig. 3:**
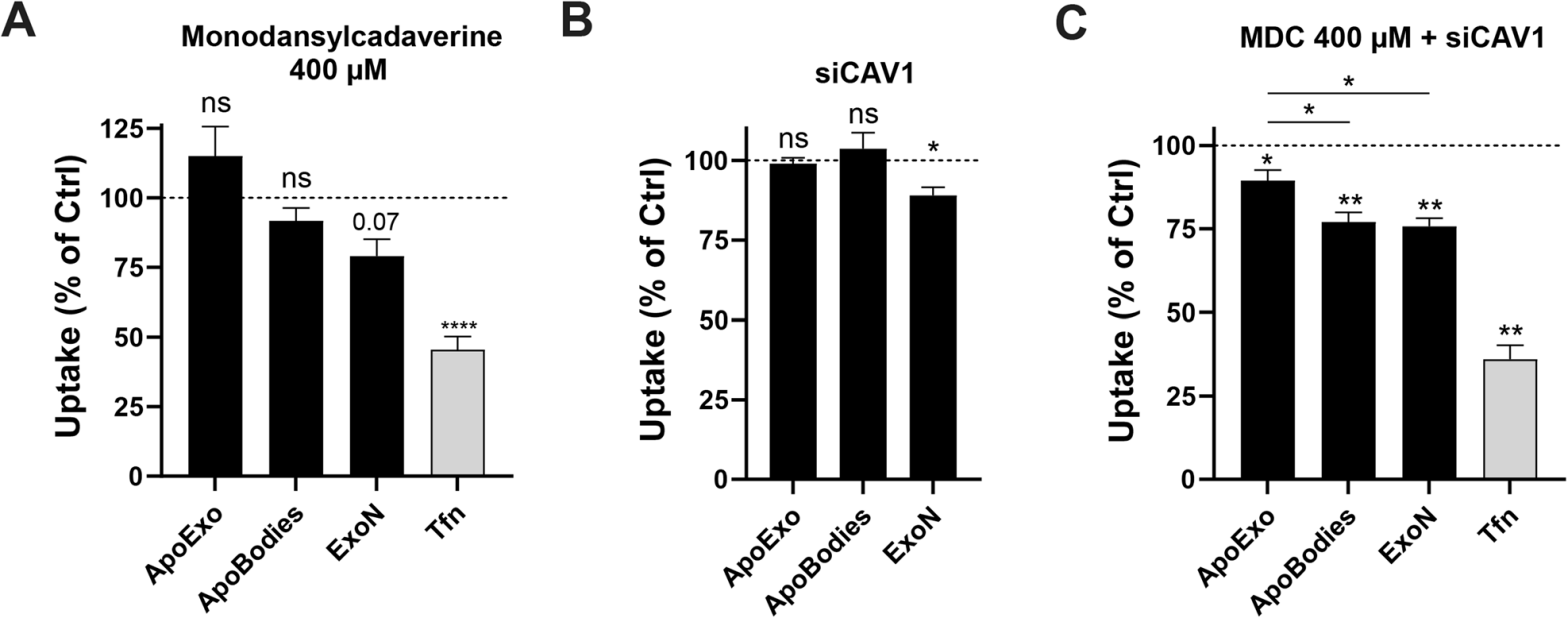
Apoptotic exosome-like vesicles are internalized by a nonclassical endocytosis pathway. **A** Quantification of ApoExo, ApoBodies, and ExoN uptake and transferrin (Tfn) uptake in serum-starved endothelial cells pretreated for 30 min with monodansylcadaverine 400 µM or its vehicle (DMSO; Ctrl) followed by a treatment of 1 h, as determined by flow cytometry. *n* = 3 for each condition. **B** Serum-starved endothelial cells were transfected with Ctrl or 90 nM CAV1 siRNA and exposed to labeled ApoExos, ApoBodies, or ExoN for 1 h. Quantification of EV uptake by flow cytometry. Representative immunoblot after CAV1 knockdown is depicted in Fig. S3. *n* = 3 for each condition. **C** Serum-starved endothelial cells were transfected with Ctrl or 90 nM CAV1 siRNA and then pretreated for 30 min with 400 µM monodansylcadaverine or a vehicle (DMSO; Ctrl) followed by a 1 h treatment with labeled ApoExos, ApoBodies or ExoNs. Quantification of EV uptake by flow cytometry. *n* = 3 for each condition. Flow cytometry experiments expressed as the cell fluorescence percentage of vehicle-treated cells MFI (30,000 events/sample) ± SEM. P values were obtained by unpaired t test (*P < 0.05, **P < 0.01, and ***P < 0.001).

Next, we sought to identify the specific cellular pathways associated with the uptake of ApoExos by endothelial cells. Classical exosomes are usually taken up by cells via endocytosis [9, 23, 24]. Clathrin-mediated endocytosis of ApoExos, ApoBodies, and ExoNs was assessed using the pharmacological inhibitor monodansylcadaverine [25]. The exposure of endothelial cells to monodansylcadaverine did not reduce the uptake rate of ApoExos or ApoBodies but did reduced transferrin, a protein known to enter cells through clathrin-dependent endocytosis, and ExoN to a near-significant trend (Fig. 3A). Caveolin-dependent endocytosis is also a well-characterized clathrin-independent endocytosis pathway. Silencing caveolin-1 in endothelial cells did not impede the uptake of ApoExos or ApoBodies but significantly impaired the uptake of ExoNs (Fig. 3B, Fig. S3). Since the outcomes of clathrin-mediated endocytosis and caveolin-dependent endocytosis may offset each other, we targeted both pathways [25]. Inhibition of both endocytic pathways resulted in a significant reduction in the uptake of ApoBodies and ExoNs and a significantly less pronounced reduction in ApoExo internalization (Fig. 3C). Taken together, these results suggest that classical endocytosis is not the main pathway for ApoExo internalization by endothelial cells.

**Fig. 4:**
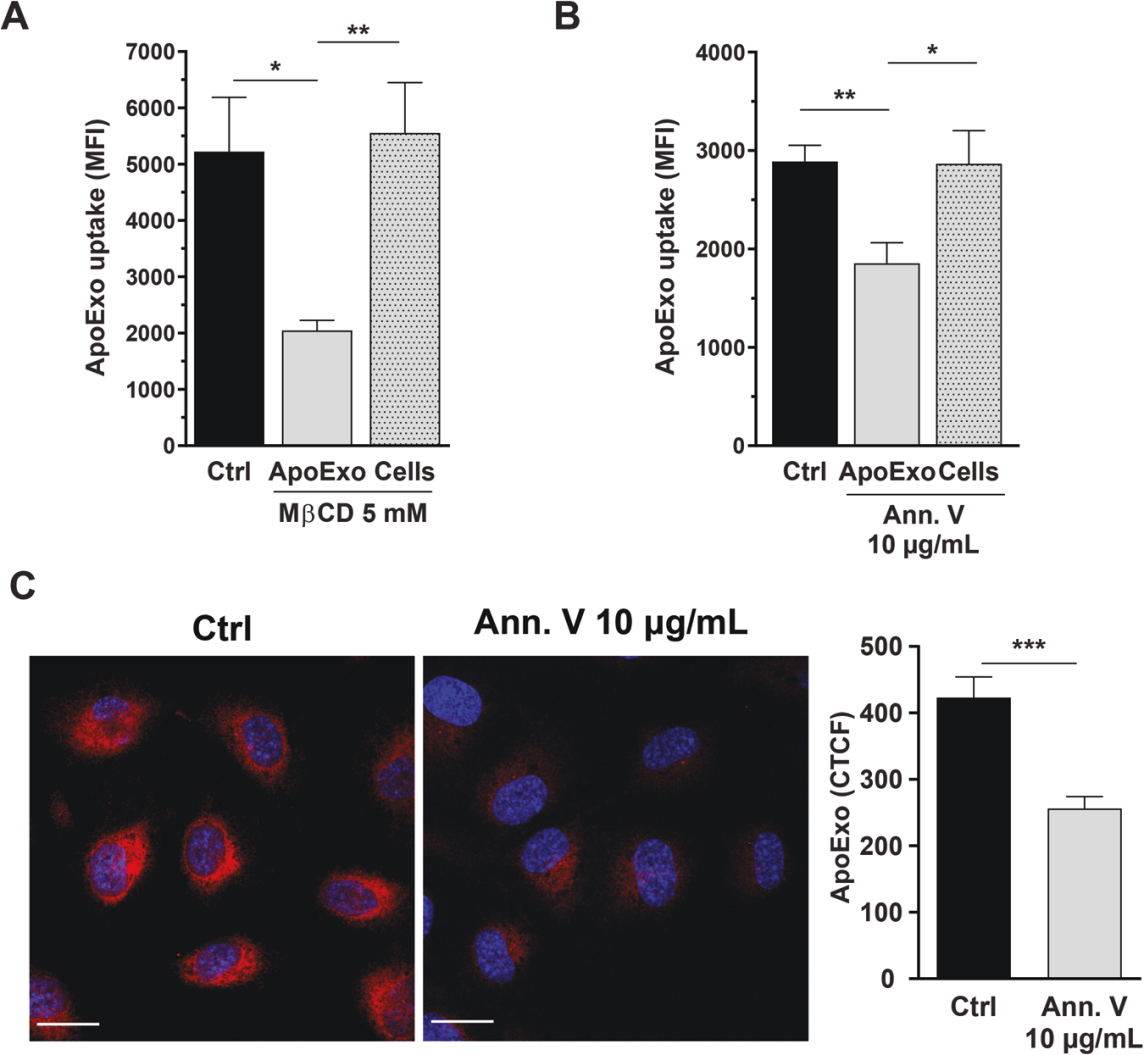
Apoptotic exosome-like vesicle internalization is mediated by a phosphatidylserine-dependent mechanism. **A** Disruption of ApoExo membrane integrity inhibits uptake. Quantification of ApoExo uptake by serum-starved endothelial cells treated with 5 mM MβCD or vehicle (water; Ctrl), as determined by flow cytometry. ApoExos were treated with 5 mM MβCD for 1 h and then incubated with serum-starved endothelial cells for 1 h, or serum-starved endothelial cells were pretreated for 1 h with 5 mM MβCD followed by incubation with ApoExos for 1 h without MβCD. *n* = 4 for each condition. Phosphatidylserine sequestration by ApoExos inhibits their uptake by endothelial cells. Quantification by flow cytometry (**B**) and confocal microscopy (**C**) of the uptake of ApoExos pretreated for 1 h with 10 µg/mL annexin V or vehicle (water; Ctrl) and then incubated with serum-starved endothelial cells for 1 h. *n* = 5 for each condition. Flow cytometry data are expressed as the median fluorescence intensity (MFI) (30,000 events/sample) ± SEM. Confocal microscopy experimental data are expressed as corrected total cell fluorescence (CTCF) ± SEM. Representative pictures of ApoExo internalization by confocal microscopy (Scale bar: 20 µm; red—ApoExo, and blue—nucleus). *P* values were obtained by unpaired t test (*P < 0.05, **P < 0.01, and ***P < 0.001).

Nonclassical endocytosis pathways were then investigated. MβCD, an inhibitor of lipid raft-mediated endocytosis, failed to block the internalization of ApoExos when used to treat endothelial cells (Fig. 4A). However, exposing ApoExos to MβCD prior to their interaction with endothelial cells significantly inhibited ApoExo uptake (Fig. 4A). This finding suggested that preservation of ApoExo membrane integrity is crucial to their internalization. Since these vesicles originate from apoptotic cells and are positive for annexin V, as described above, we evaluated the importance of phosphatidylserine to their internalization. ApoExos coated with annexin V, a protein with high affinity for phosphatidylserine [26], showed reduced uptake by endothelial cells. However, when endothelial cells, but not ApoExos, were exposed to annexin V prior to cell interaction with ApoExos, no reduction in the uptake rate was observed (Fig. 4B, C). These results suggests that phosphatidylserine on the surface of ApoExos is a key element controlling their uptake by endothelial cells.

### Endothelial cells internalize apoptotic exosome-like vesicles through macropinocytosis

**Fig. 5:**
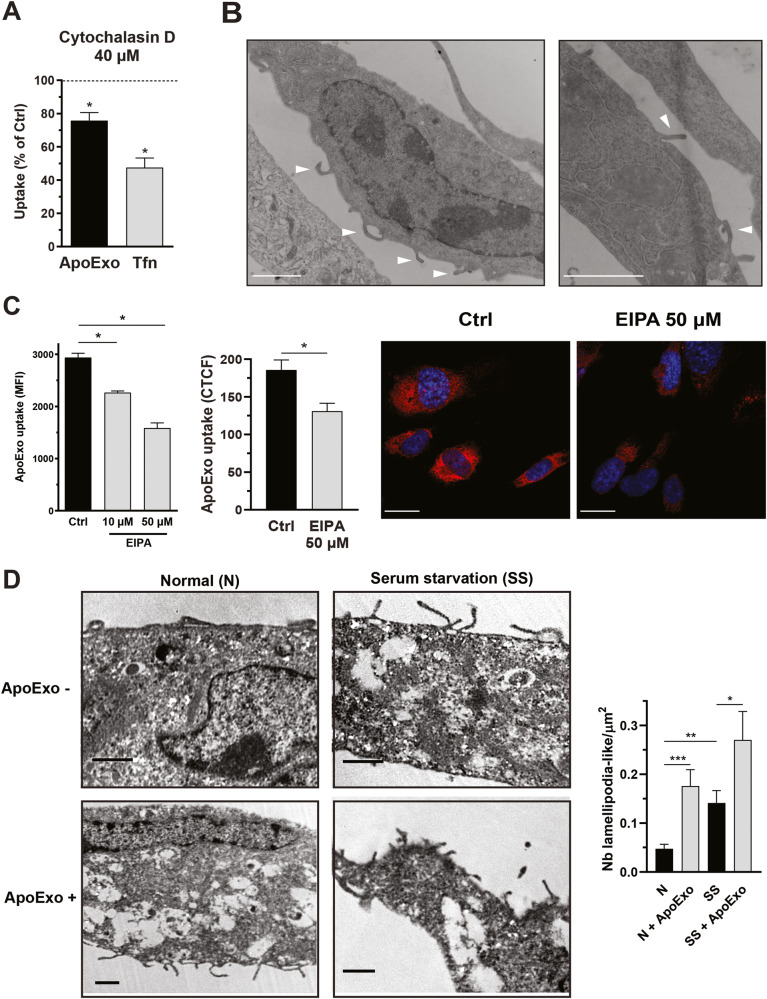
Apoptotic exosome-like vesicles are internalized by endothelial cells through macropinocytosis. **A** Cytoskeleton dynamics inhibition suppressed ApoExo uptake by endothelial cells. Quantification of ApoExo uptake and transferrin (Tfn) uptake by flow cytometry in serum-starved endothelial cells pretreated for 30 min with 40 µM cytochalasin D or vehicle (DMSO; Ctrl) followed by treatment for 1 h. *n* = 3 for each condition. **B** Endothelial cells displayed macropinosome formation. Representative electron micrograph showing macropinosome formation (arrow) in endothelial cells after serum starvation for 2 h. Scale bar: 2 µm, *n* = 3. **C** Macropinocytosis inhibition suppressed ApoExo uptake by endothelial cells. Quantification of Apex uptake in serum-starved endothelial cells pretreated for 30 min with 10 and 50 µM EIPA or its vehicle (DMSO; Ctrl) followed by treatment for 1 h as determined by flow cytometry (left) and confocal microscopy (right) in. *n* ≥ 3 for each condition. Flow cytometry data are expressed as the median fluorescence intensity (MFI) (30,000 events/sample) ± SEM. Confocal microscopy experiment data are expressed as corrected total cell fluorescence (CTCF) ± SEM. Representative pictures of ApoExo internalization by confocal microscopy. (Scale bar: 20 µm; red—ApoExo and blue—nucleus). **D** ApoExo treatment induced lamellipodium-like structure formation in both normal (N) and serum-starved (SS) cells after 1 h. *n* ≥ 5 for each condition from a single experiment. Representative electron micrographs showing an increased number of lamellipodium-like structures for each condition. Macropinocytosis is observed following invagination of the cell membrane by mobilization of actin filaments to form protrusions that fold over the plasma membrane to form a macropinosome. TEM images show the number of structures per cell surface ± SEM. Scale bar: 1 µm. *P* values were obtained by unpaired t test (*P < 0.05, **P < 0.01, and ***P < 0.001).

Next, we wanted to characterize the endocytic pathway in endothelial cells that mediate ApoExo internalization. Endothelial cells trigger various mechanisms to internalize extracellular components, including macropinocytosis [27]. The macropinocytosis pathway is associated with the actin-dependent formation of membrane ruffles and results in the uptake of fluid into large vacuoles [28]. Disrupting the actin cytoskeleton by exposing endothelial cells to cytochalasin D significantly attenuated ApoExo uptake, confirming the importance of cytoskeletal functional integrity for ApoExo internalization (Fig. 5A). We then used ultrastructural analysis via electron microscopy to study the dynamics of macropinocytosis in resting endothelial cells and endothelial cells actively engaged in internalizing extracellular material. Macropinocytosis was observed in endothelial cells at baseline in electron micrographs, which showed membrane ruffling and lamellipodium-like structures (Fig. 5B). To further investigate the role of macropinocytosis in ApoExo uptake, endothelial cells were exposed to ethyl isopropyl amiloride (EIPA), a potent macropinocytosis inhibitor [29]. Inhibition of macropinocytosis significantly reduced the internalization of ApoExos by endothelial cells (Fig. 5C). In addition, the EIPA-induced inhibition of EV uptake was significantly greater for ApoExos and apoptotic bodies than for normal exosomes (Fig. S4A). Importantly, this effect was not restricted to HUVECs, as EIPA similarly inhibited ApoExo uptake by mPRECs and WI38 fibroblasts (Fig. S2C). Next, we tested whether the combined inhibition of phosphatidylserine and macropinocytosis resulted in the synergistic inhibition of ApoExo internalization. Blocking phosphatidylserine from exposure on the surface of ApoExo did not enhance the inhibition induced by macropinocytosis disruption (Fig. S4B). This suggested that phosphatidylserine-dependent signals and macropinocytosis function through the same pathway to regulate the uptake of ApoExos. Since several agonists, viruses and bacteria can induce macropinocytosis to induce their own uptake, we assessed the ability of ApoExos to induce a positive feedback loop to promote macropinocytosis thus increase their own internalization. Endothelial cells exposed to ApoExos showed an increase in lamellipodium-like structures compared to cells under either normal or serum-starved conditions (Fig. 5D). These results indicate that phosphatidylserine-dependent macropinocytosis is instrumental in the internalization of ApoExos and that in turn, ApoExos can promote a positive feedback loop favoring their internalization by increasing the macropinocytic activity of endothelial cells.

### Apoptotic exosome-like vesicles transfer RNA to endothelial cells through macropinocytosis

**Fig. 6:**
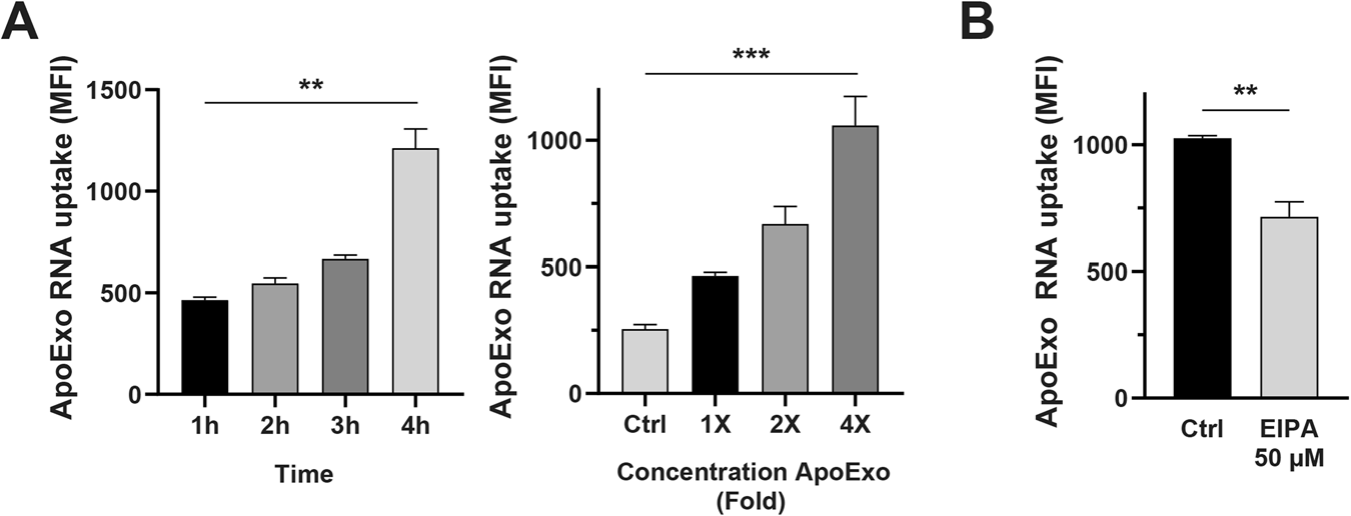
ApoExos transfer RNA to endothelial cells by macropinocytosis. **A** Quantitative measurement of RNA-labeled ApoExo uptake by flow cytometry. Left graph: Time-dependent uptake of RNA-labeled ApoExo by endothelial cells under serum starvation from 1 to 4 h. Right graph: Concentration-dependent uptake of RNA-labeled ApoExos by serum-starved endothelial cells during 1 h. Control (Ctrl) represents cells treated with RPMI serum-free medium. *n* ≥ 3 for each condition. **B** Macropinocytosis inhibition suppressed ApoExo RNA uptake by endothelial cells. Quantification of Aphex uptake by serum-starved endothelial cells pretreated for 30 min with 50 µM EIPA or its vehicle (DMSO; Ctrl) followed by treatment for 1 h, as determined by flow cytometry. *n* ≥ 3 for each condition. Flow cytometry data are expressed as the median fluorescence intensity (MFI) (30,000 events/sample) ± SEM. *P* values were obtained by unpaired t test (*P < 0.05, **P < 0.01, and ***P < 0.001).

Classical exosomes have been previously shown to function as cargos for intercellular sharing of mRNA and microRNA [30, 31]. ApoExos exhibit a pattern of mRNA and microRNA expression distinct from those of apoptotic bodies and classical exosomes [19]. To ascertain whether the uptake of ApoExo by endothelial cells leads to intercellular transfer of nucleic acids, we labeled the RNA content of ApoExos. The uptake of RNA-labeled ApoExos showed similar dynamics to that of ApoExos labeled with the protein dye CRMA (Fig. 6A). Endothelial cells internalizing ApoExos treated with RNAse emitted a similar intensity of fluorescence as emitted by cells exposed to untreated ApoExos. As RNAse degrades any significant free RNA in an ApoExo suspension, failure to reduce cell uptake of RNA in RNAse-treated ApoExos confirmed that RNA transfer resulted from ApoExo internalization (Fig. S5). We then asked, is macropinocytosis also a key mechanism for the uptake of RNA cargo carried by ApoExos? We answered this question by inhibiting macropinocytosis in endothelial cells exposed to RNA-labeled ApoExos. Macropinocytosis inhibition with EIPA reduced the uptake of RNA-labeled ApoExos (Fig. 6B), demonstrating a central role for macropinocytosis in intercellular RNA transfer.

**Fig. 7:**
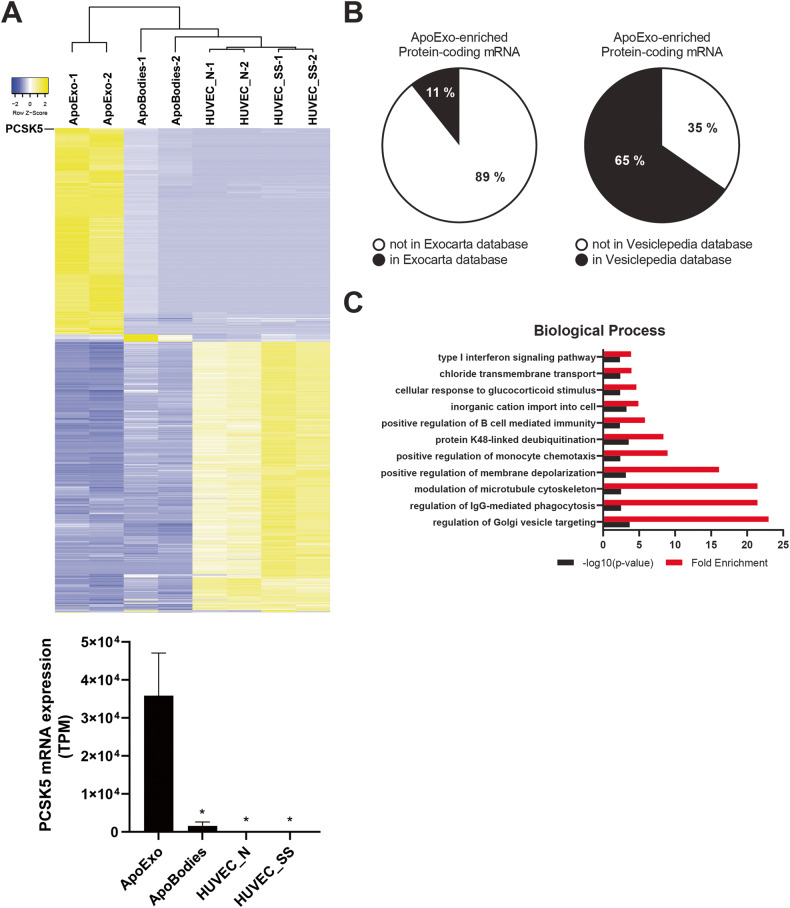
ApoExos show a specific profile of mRNAs. **A** Heatmap representation of protein-encoding RNA sequences and PCSK5 mRNA expression (TPM; transcripts per million) in ApoExos, ApoBodies, and endothelial cells under normal (HUVEC_N) or serum-starved (HUVEC_SS) conditions; *n* = 2. **B** Analysis of the percentage of mRNA identified in the transcriptome of ApoExos and present in the ExoCarta or Vesiclepedia databases. **C** Gene Ontology analysis of biological processes for mRNAs enriched in ApoExos (Table S2). Terms related to membrane depolarization, microtubule cytoskeleton, IgG-mediated phagocytosis, and vesicle targeting were enriched.

Exosomes have been shown to carry functional mRNA cargos to recipient cells [30, 32], thus regulating protein expression and cell function. We assessed whether ApoExos can carry functional mRNAs, which would lead to mRNA expression in the endothelial cells that internalize them. We therefore sequenced RNA extracted from ApoExos, apoptotic bodies, and endothelial cells exposed to serum starvation or maintained under normal conditions. Hierarchical clustering and principal component analysis (PCA) using protein-coding mRNAs in extracellular vesicles and cells were performed (Fig. 7A, Fig. S6A). The analysis confirmed that ApoExos expressed a specific mRNA profile that differed from that of apoptotic bodies and parental endothelial cells under either serum starved or under normal conditions. Among the 430 mRNAs identified in ApoExos (Table S1), only 11 and 65% of the ApoExo transcriptomes were listed in the ExoCarta and Vesiclepedia databases, respectively (Fig. 7B), further supporting the notion that ApoExos differ from apoptotic bodies and present a distinct mRNA profile compared to classical exosomes. We then performed a Gene Ontology analysis to further insights into the biological processes potentially modulated by ApoExo-enriched mRNAs (Fig. 7C, Fig. S6B, Tables S2–4). Biological processes related to regulation of membrane depolarization, microtubule cytoskeleton, IgG-mediated phagocytosis and vesicle targeting were enriched in ApoExos.

**Fig. 8:**
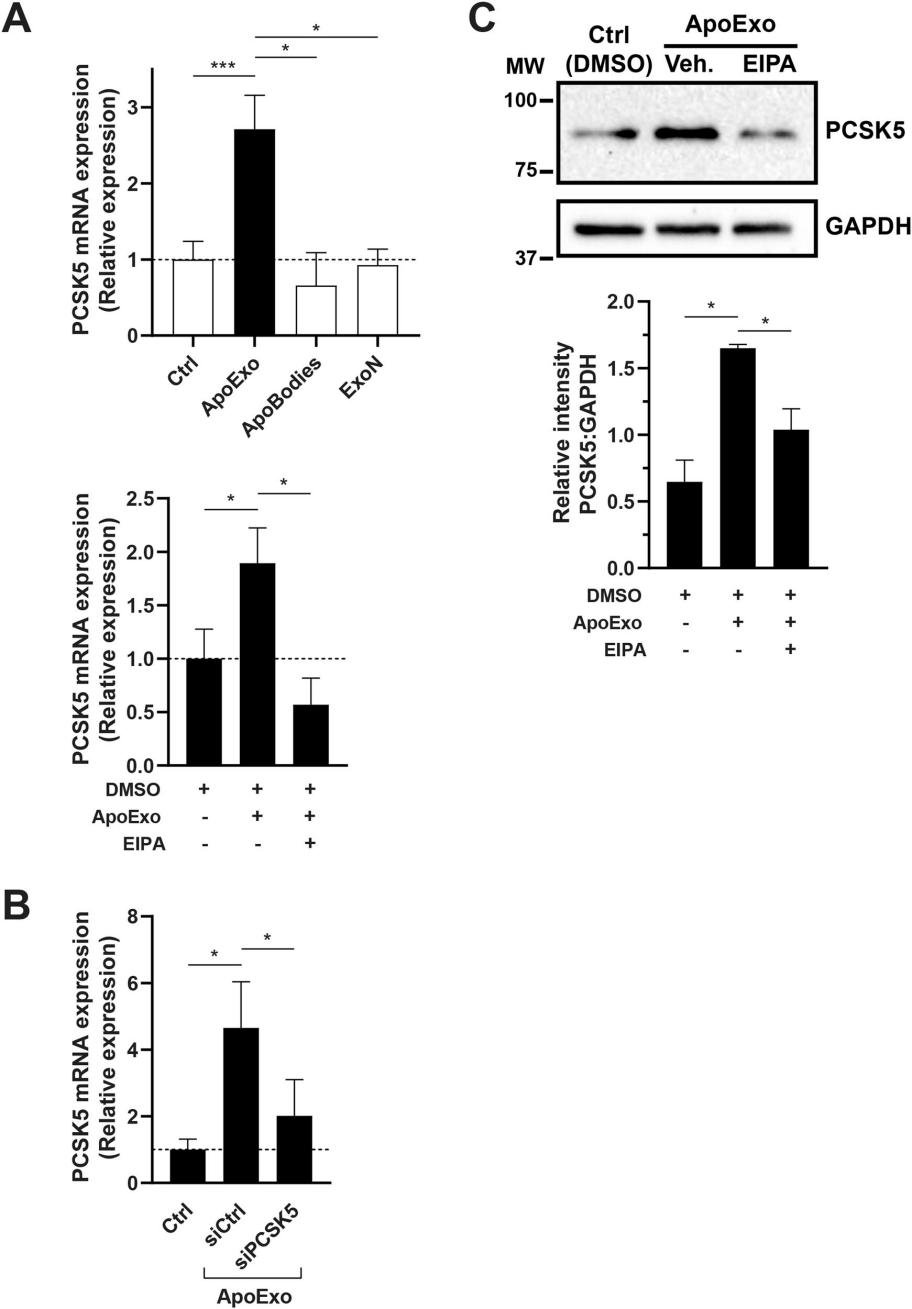
ApoExos transfer functional PCSK5 mRNA to endothelial cells, increasing cellular PCSK5 protein levels. **A** ApoExo uptake through macropinocytosis increases PCSK5 mRNA expression in endothelial cells. Endothelial cells were treated with ApoExos, ApoBodies, or ExoN for 24 h (upper graph) or exposed to ApoExos and DMSO or ApoExos and EIPA 30 µM for 24 h (lower graph). Control (Ctrl) represents cells treated with RPMI serum-free medium. *n* ≥ 3 for each condition. **B** Knockdown of PCSK5 in ApoExos inhibits the increase in PCSK5 mRNA expression in recipient endothelial cells. Endothelial cells were treated with 90 nM ApoExos isolated from endothelial cells transfected with Ctrl (siCtrl) or PCSK5 (siPCSK5) siRNA; *n* ≥ 3 for each condition. Expression of PCSK5 mRNA was measured by quantitative RT‒PCR, and the result is presented as relative expression of PCSK5 mRNA compared to untreated cells (Ctrl) ± SEM after normalization with HPRT1. **C** Macropinocytosis-dependent ApoExo uptake increased PCSK5 protein expression. Endothelial cells were exposed to vehicle (Ctrl - DMSO), ApoExos with vehicle (ApoExos + DMSO), or ApoExos + EIPA 30 µM (ApoExos + EIPA) for 24 h. PCSK5 expression was quantified by densitometry and expressed as arbitrary units ± SEM; *n* ≥ 3. Cropped representative images of immunoblots from the same gel are presented. *P* values were obtained by unpaired t test (*P < 0.05, **P < 0.01, and ***P < 0.001).

Among mRNAs specifically enriched in ApoExos, PCSK5 was the most enriched protein-encoding RNA (Fig. 7A, Table S1). We treated endothelial cells with ApoExos or controls followed by a wash-out to avoid nucleic acid contamination from suspended ApoExo, then we measured PCSK5 mRNA levels. Endothelial cells treated with ApoExos, but not with classical exosomes, apoptotic bodies, or EV-depleted conditioned medium, showed significantly increased PCSK5 mRNA levels. Inhibition of macropinocytosis with EIPA in endothelial cells prevented the upregulation of PCSK5 mRNA in endothelial cells exposed to ApoExos (Fig. 8A, Fig. S7A). Next, we confirmed that the increase in PCSK5 mRNA expression induced by ApoExos was indeed mediated by the direct transfer of PCSK5 mRNA and not by the modulation of PCSK5 gene expression in recipient cells. Endothelial cells exposed to ApoExos derived from endothelial cells and depleted of endogenous PCSK5 mRNA (Fig. S7B) did not exhibit increased PCSK5 mRNA expression (Fig. 8B). Intercellular mRNA transfer does not necessarily lead to changes in protein levels, as transferred nucleic acids can be degraded and thus not translated. We then tested whether endothelial cells exposed to ApoExos show increased PCSK5 protein levels. ApoExos induced a significant increase in PCSK5 protein levels in endothelial cells, which was prevented by treatment with a macropinocytosis inhibitor (Fig. 8C, Fig. S7C). As ApoExos did not express PCSK5 protein but only PCSK5 mRNA (Fig. S7D), these findings suggest that ApoExos transfer specific functional RNAs to endothelial cells, thereby regulating cell protein levels.

## Discussion

Apoptosis has long been appreciated as a mechanism leading to the production of apoptotic bodies whose engulfment by professional phagocytes contributes to the activation of anti-inflammatory and tolerogenic pathways [33, 34]. ApoExos, also produced by apoptotic cells, have been characterized more recently, and little is known about the mechanisms of ApoExo uptake. Although ApoExos are similar exosomes, which are known to be taken up by a wide variety of cell types, ApoExos display distinct features that differentiate them from both apoptotic bodies and classical exosomes. ApoExos form in stressed cells within large autolysosomes and are released into the extracellular milieu through caspase-3-dependent fusion of autolysosomes with the cell membrane [16]. However, whether ApoExos can be internalized by endothelial cells to transfer nucleic acids from dying cells to neighboring cells and whether nucleic acid sharing can impact protein levels in cells that internalize them remained to be delineated. In the present study, we demonstrated that ApoExos expressed distinct protein and mRNA profiles compared with other types of extracellular vesicles and showed that endothelial cells actively internalized ApoExos through phosphatidylserine-dependent macropinocytosis. mRNAs specifically enriched within ApoExos, such as PCSK5, were taken up by endothelial cells, and blocking macropinocytosis prevented PCSK5 uptake. These results demonstrate that mRNAs specifically enriched in ApoExos can be delivered to cells via ApoExo internalization, thereby impacting cell protein expression. As we showed that ApoExos exhibited a distinctive set of mRNAs compared to other extracellular vesicles, these results suggest a distinct role for ApoExos in shaping endothelial protein expression and, therefore, endothelial function. We also found that ApoExos promoted macropinocytic activity in endothelial cells, suggesting that they can induce a positive feedback loop for increasing ApoExo entry and cargo delivery.

Classical endocytosis is the main pathway for EV uptake. Various pathways are involved in the internalization of exosomes, and the best-characterized pathways are the clathrin and caveolin-dependent endocytic pathway [35]. Although the contribution of macropinocytosis to extracellular vesicle internalization has been increasingly recognized, macropinocytosis is generally considered a pathway that complements clathrin and caveolin-dependent endocytosis [36–39]. Here, we not only demonstrated that classical endocytosis pathways were not involved in ApoExo internalization but also showed that macropinocytosis was the central pathway for ApoExo uptake. Our results provide new insights into the mechanisms of ApoExo uptake, which further support the notion that ApoExos differ from other types of extracellular vesicles. While both apoptotic bodies and ApoExos are internalized via phosphatidylserine-dependent cellular pathways, apoptotic bodies are cleared by professional phagocytes via opsonization initiated by phosphatidylserine receptors, including the TIM superfamily receptors [40, 41]. Whether ApoExo internalization follows a similar pathway or is independent of receptors remains to be determined. However, the inability to completely inhibit ApoExo internalization by blocking macropinocytosis suggests that other pathways contribute to ApoExo uptake.

Macropinocytosis is a constitutive cellular process through which nonspecific extracellular contents, such as soluble molecules and nutrients, are engulfed by cells. Recent evidence suggests that macropinocytosis is involved in other cellular processes, such as antigen presentation by professional antigen-presenting cells (APCs), including dendritic cells and macrophages [42], and nonprofessional phagocytes, such as endothelial cells [43]. Recent evidence also suggests that exosomes participate in antigen presentation [44, 45] by transferring antigenic peptides to the APCs that internalize them. Our results describing the predominant role of macropinocytosis in ApoExo uptake suggest the possibility that macropinocytosis regulates the known autoimmune activity of ApoExos. Indeed, ApoExos carry different autoantigens, including LG3, a fragment of perlecan, and promote the production of autoantibodies, including anti-LG3 autoantibodies, which have been associated with vascular inflammation and kidney dysfunction in mice and human renal transplant recipients [13, 15, 16, 46–48]. We also showed that ApoExos leverage macropinocytosis to foster their own internalization by recipient cells. This action is also found in many pathogens, such as viruses, which activate signaling pathways that trigger membrane ruffling [49, 50]. Our findings suggest that the production of ApoExos at sites of vascular injury may trigger macropinocytotic activity, rendering vascular beds potentially more vulnerable to viral entry. The molecular effectors of macropinocytosis that regulate the uptake of ApoExos and the implications of these findings on virus infection dynamics will be assessed in future studies.

ApoExos, similar to classical exosomes, carry nucleic acid cargoes involved in intercellular communication. While most studies on exosomes have focused on miRNAs, we showed that ApoExos are not only enriched in immunogenic RNA [19] but also exhibit a unique protein-coding RNA profile. Indeed, a majority of the mRNAs identified in ApoExos are not listed in the ExoCarta or Vesiclepedia databases. Interestingly, biological processes activated by these RNAs included ion channel regulation, membrane depolarization, and cytoskeleton reorganization, which are central to macropinocytic activity [51]. ApoExos can efficiently cross the cell membrane, and their RNA content is thus delivered in the cytoplasm. Internalization of exosomes by macropinocytosis results in the delivery of vesicular cargo to the endosomal pathway. Early endosomes undergo sorting before transitioning to late endosomes and ultimately degrade exosomes after endosome fusion with lysosomes [52]. Here, we show that PCSK5 mRNA, the most prominent mRNA found to be carried by ApoExos, escaped degradation in recipient cells, leading to increased protein levels. Further studies are needed to characterize the intracellular trafficking pathways following the uptake of ApoExos by recipient cells.

PCSK5, a serine protease of the proprotein convertase family, posttranslationally activates precursor proteins into their active forms via specific cleavage. The role of PCSK5 expressed following ApoExo uptake by recipient cells remains to be determined. PCSK5 may potentially contribute to the acquisition of migration-promoting phenotype in endothelial cells [18] and vascular remodeling in a murine models of allograft rejection [13, 53] induced by ApoExos through matrix metalloproteinase activation, extracellular matrix remodeling, or collagen deposition [54–56]. Recently, PCSK5 has been shown to be involved in the generation of urinary peptides associated with the progression of chronic kidney disease [57]. Previously, we showed that apoptosis of endothelial cells leading to rarefaction of peritubular capillaries was a key determinant of chronic kidney disease [58–60]. Apoptosis of endothelial cells, whether at the time of ischemia‒reperfusion injury or during ongoing injury, triggered the release of ApoExos downstream of caspase-3 activation [13, 16]. Whether ApoExos and PCSK5 contribute to the transition from acute kidney injury to chronic kidney disease will be addressed of future studies.

**Fig. 9:**
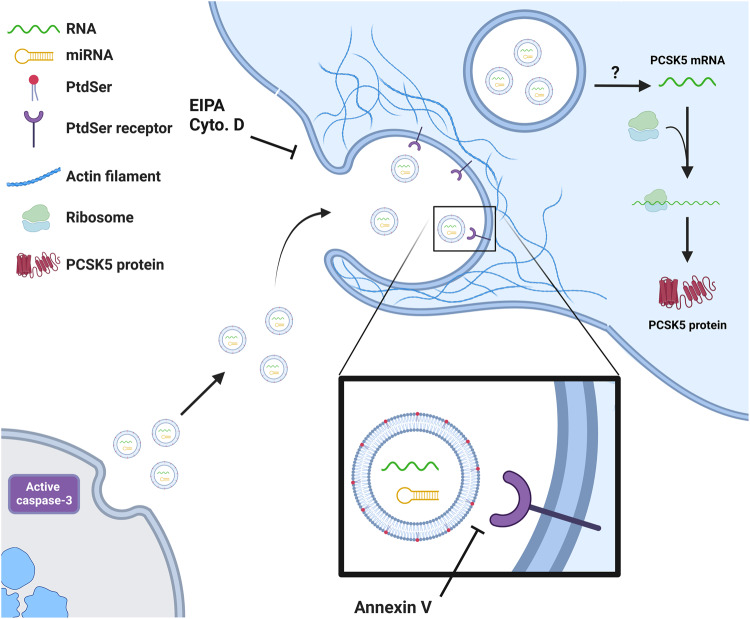
ApoExos transfer functional mRNAs to endothelial cells via phosphatidylserine-dependent macropinocytosis. ApoExo uptake depends on phosphatidylserine-dependent macropinocytosis, which is further enhanced by ApoExos, which induce an increase the macropinocytic activity in endothelial cells. Once internalized, PCSK5 mRNA, the most abundant mRNA in ApoExos, escapes lysosomal degradation to increase PCSK5 protein expression in endothelial cells. The schematic was created with BioRender.com.

Taken together, these results suggest that ApoExos represent a unique way for injured cells to transfer active and specific mRNA cargo to neighboring endothelial cells. ApoExo uptake depends on phosphatidylserine-dependent macropinocytosis, which is further enhanced by the ApoExos themselves. PCSK5, the most abundant mRNA carried by ApoExos, is induced to expression after uptake endothelial cells, leading to increased protein expression (Fig. 9). Collectively, these results show ApoExos as novel regulators of endothelial function and protein expression at sites of vascular injury.

## Materials and methods

### Cell culture and reagents

Human umbilical vein endothelial cells (HUVECs) were purchased from Cell Applications, cultured in Medium 200 + LSGS (Gibco, Waltham, MA, USA) on a gelatin-coated surface, and Passage 4 cells were used in experiments. Endothelial cells were exposed to RPMI serum-free medium (Gibco) or Medium 200 + LSGS (depleted of vesicles) for 4 h to produce apoptotic-cell- or healthy-cell-conditioned medium respectively as described previously [13, 15, 18, 19]. To generate fluorescence-emitting vesicles, HUVECs were stained using CellTracker Orange-CMRA dye or SYTO RNASelect Green Fluorescent cell stain (Molecular Probes, Waltham, MA, USA) for 15 min before treatment. Cytochalasin D, monodansylcadaverine, methyl-β-cyclodextrin (MβCD), and 5-(N-ethyl-N-isopropyl)amiloride (EIPA) were purchased from Sigma (Burlington, MA, USA). FITC-Transferrin was obtained from Thermo Fisher (Waltham, MA, USA), and annexin V was obtained from BioLegend (San Diego, CA, USA).

### Extracellular vesicle isolation

Apoptotic-cell or healthy-cell conditioned medium from HUVEC cultures was fractionated using sequential centrifugation as described previously [13, 15, 18, 19]. Briefly, a centrifugation at 1200 × *g* was first performed for 15 min at 4 °C to pellet cell debris; then centrifugation was performed at 50,000 × *g* for 15 min at 4 °C to pellet apoptotic bodies; and finally ultracentrifugation was performed at 200,000 × *g* for 18 h at 4 °C to pellet apoptotic exosome-like vesicles. ApoExos were purified from serum-free medium conditioned from cultures of 4.9 × 10^6^ HUVECs and resuspended in an initial volume of conditioned medium (25 mL) for further experiments. We submitted all relevant data related to our experiments to the EV-TRACK knowledgebase (EV-TRACK ID: EV220401) [61].

### Apoptosis level assessment

HUVECs were grown in 6-well plates until they reached 90–95% confluence. The cells were then washed twice with RPMI before incubation in either RPMI or complete M200 medium for 4 h. The cells were then stained with Hoechst33342 (1:10,000) (Molecular Probes, Waltham, MA, USA) for 10 min. Propidium iodine solution was added to the culture medium (final concentration 5 μg/mL) immediately before analysis (excitation filter, λ = 360–425 nm). Normal, apoptotic, and necrotic adherent cell proportions in eight random fields per condition were assessed by an investigator blinded to the experimental conditions. Apoptotic cells with condensed nucleus (bright blue) in the absence of cell membrane permeability (red) were counted, and the number of apoptotic cells was divided by the total number of cells in each micrograph to determine the percentage of apoptotic cells.

### Proteasome activity assay

Isolated ApoExos and apoptotic bodies were resuspended at a concentration of 200X in PBS, and the protein concentration was assessed using a BCA microassay kit (Thermo Fisher, Waltham, MA, USA). Then, a proteasome caspase-like activity assay kit was used according to the manufacturer’s instructions (Promega, Madison, WI, USA) to assess proteasome activity. Briefly, ApoExo or apoptotic body resuspensions were diluted in PBS until the total protein concentrations and total volumes were equal, and the suspension were heated to room temperature. Luminescent reagent was then added to the sample in equal volume, covered with aluminum foil to protect from light, and agitated at 200 rpm on an orbital shaker. Samples were incubated at room temperature for 10 min before reading with a Victor^3^ luminescence plate reader (PerkinElmer, Waltham, MA, USA). The luminescence levels were normalized to the protein levels and reported as activity per initial volume of supernatant.

### Flow cytometric analyses of extracellular vesicles

Cells were cultured in vesicle-depleted medium 200 + LSGS or RPMI serum-free medium, as described in the previous section. Harvested supernatants were centrifuged at 1200 × *g* for 15 min at 4 °C to remove cell debris. Extracellular vesicles were labeled for 60 min at 32 °C with a LWA300 proteasome probe (300 nM). Cell Tracker Deep Red (1 µM, Thermo Fisher) was added and incubated with the EVs for 30 min at 32 °C, and then, 1 μL of Annexin V-BV421 (BD Biosciences) was added to the EVs, and then, the mixture was incubated for 20 min at room temperature. Flow cytometric analyses were performed as previously described on a BD Canto II Special Order Research Product (BD Biosciences) equipped with a small particle option [16].

### Flow cytometry

Confluent cells were incubated with labeled ApoExos for the indicated times. Cells were washed in PBS, detached with trypsin, subsequently incubated with an AquaDead cell stain kit (Invitrogen, Waltham, MA, USA) according to the manufacturer’s instructions, and washed twice in PBS supplemented with 1% BSA (w/v). ApoExo internalization was analyzed by flow cytometry with a FACS-LSRII instrument integrated with FACSDiva software (BD Biosciences, Franklin Lakes, NJ, USA). The data were analyzed using FlowJo software. Graphs show median fluorescence values (30,000 events/sample).

### Confocal microscopy

The following protocol was adapted from Migneault et al. [18]. Cells were grown on gelatin-coated glass coverslips until confluence and incubated with labeled ApoExos for the indicated periods. The cells were washed and fixed using 4% (w/v) paraformaldehyde in PBS for 20 min at room temperature. Fixed cells were stained with DAPI (300 nM) in PBS and mounted on glass microscope slides using Prolong Gold mounting reagent. Confocal images were acquired with a Leica TCS-SP5 inverted microscope using an HCX PL APO 63x/1.4 oil objective. An excitation system was established using a 405-nm diode laser for exciting DAPI and a 561-nm diode laser for exciting CMRA-stained vesicles, and sequential acquisition was performed at a scan speed of 400 Hz. Images were acquired with Las-AF software. The final images were 12 bits, 2048 × 2048 with a zoom factor of 2; the scale is specified in figure legends. Images were analyzed using FIJI software (NIH). The corrected total cell fluorescence (CTCF) represents the integrated density minus an area of the selected cell multiplied by the mean fluorescence of the background readings [18, 62].

### Electron microscopy

HUVECs were fixed with 1% glutaraldehyde in phosphate buffer, pH 7.2. The cells were washed and scraped off the plates in phosphate buffer and pelleted. The pellet was postfixed with 1% OsO_4_ in phosphate buffer for 1 h at 4 °C and then dehydrated in a graded series of ethanol and embedded in Epon according to routine techniques (Luft 1961). Ultrathin sections were obtained using a Reichert Ultracut ultramicrotome and mounted on naked nickel grids. Sections were stained with 3% aqueous uranyl acetate and lead citrate, and examination was performed with a Philips CM100 transmission electron microscope. Electron micrographs were captured using an AMT XR80 digital camera.

### siRNA transfection

This protocol was adapted from Migneault et al. [18]. Cells were plated onto 6-well plates at 2500 cells per cm^2^. After 72 h, cells were transfected with ON-TARGETplus nontargeting siRNA #3 – SMARTpool, ON-TARGETplus Human CAV1 siRNA (90 nM) – SMARTpool, ON-TARGETplus Human PCSK5 siRNA (90 nM) – SMARTpool (Dharmacon, Lafayette, CO, USA) using Magnet Assisted Transfection (MATra) (IBA Lifesciences, Göttingen, Germany) according to the manufacturer’s instructions. Briefly, MATra-si Reagent was added to siRNA at a ratio of 1 µL:1 µg in Opti-MEM medium (Gibco) and incubated for 25 min at room temperature. The siRNA-bead mixture was added to the supernatant (Opti-MEM) of each well. Then, the plate was placed on a magnetic plate for 15 min, and the medium was changed after 30 min. After 48 h, the cells were processed according to the assay performed.

### Cell lysis, protein isolation, and immunoblotting

The following protocol was adapted from Migneault et al. [63]. Total extracted protein was obtained by washing cells twice in ice-cold PBS and lysing them for 15 min under agitation at 4 °C in lysis buffer (1% Triton X-100, 150 mM NaCl, 5 mM EDTA, and 50 mM Tris, pH 7.5) supplemented with protease inhibitor (Calbiochem, San Diego, CA, USA) and phosphatase inhibitor cocktails (Sigma). The cells were subsequently scraped with a rubber policeman, collected, and centrifuged at 12,000 × *g* for 10 min at 4 °C. The protein concentration was evaluated using a BCA protein assay kit (Thermo Fisher) according to the manufacturer’s instructions. The proteins were solubilized in sample buffer (25 mM Tris·HCl, pH 6.8, 1% SDS, 0.1% bromophenol blue, 10% glycerol, and 2% β-mercaptoethanol), incubated at 95 °C for 10 min, subjected to SDS-polyacrylamide gel electrophoresis, and transferred electrophoretically onto nitrocellulose membranes. The membranes were blocked with 5% dried fat-free milk in Tris-buffered saline at pH 7.4 with 0.05% Tween 20 (TBST) for 1 h at room temperature and then incubated overnight at 4 °C with the following primary antibodies: anti-caveolin 1 (Cell Signaling Technology), anti-dynamin I/II (Cell Signaling Technology), anti-PCSK5 (ThermoFisher), anti-GAPDH (Cell Signaling Technology) or anti-β-actin (Sigma) in TBST plus 5% milk or 5% BSA. After being washed with TBST, the membranes were incubated with goat anti-rabbit or goat anti-mouse IgG linked to horseradish peroxidase (GE Healthcare, Chicago, IL, USA) for 1 h. After washing with TBST, the membranes were incubated with Lumi-Light Western blot substrate (Roche, Basel, Switzerland) or ECL Prime Western blot detection reagent (GE Healthcare) for 5 min before the luminescence signal was recorded using a ChemiDoc XRS+ system (Bio-Rad Laboratories Inc.). The intensity of each specific band was quantified with Image Lab 5.2 software (Bio-Rad Laboratories Inc).

### RNA-Seq data

RNA-Seq data from Hardy et al. [19] were downloaded from Gene Expression Omnibus archives under accession number GSE119108. Reads were quantified and aligned on ENSEMBL annotated transcripts (GRCh38.91) using the Kallisto (v0.43.1) algorithm. Transcript expression levels are expressed as transcripts per million (TPM). To identify enriched protein-coding mRNA in ApoExo, we considered transcripts with a relative expression higher than 10 TPM in two replicates. We defined a transcript as enriched when it exhibited an expression fold change >3 in ApoExos compared to that in all three other conditions (ApoBodies, endothelial cells in normal or serum-starved condition) in both replicates. Differentially expressed mRNAs were considered significant by two-way ANOVA and Fisher’s LSD post hoc test with *P* < 0.05. An overrepresentation analysis was performed using the WEB-based GEne SeT AnaLysis Toolkit.

### Quantitative RT‒PCR

Total RNA was obtained by washing cells twice in ice-cold PBS and extracted using a miRNeasy Mini Kit (Qiagen, Hilden, Germany) according to the manufacturer’s protocol. RNA was quantified using a DS-11 Series Spectrophotometer/Fluorometer (DeNovix, Wilmington, DE, USA). One microgram of total RNA was pretreated with RNAse free-DNAse I (Invitrogen, Waltham, MA, USA) and reverse-transcribed to cDNA with iScript Reverse Transcription Supermix (Bio-Rad, Hercules, CA, USA) according to the manufacturer’s instructions. For qPCR amplification, 5 ng of cDNA was amplified with PCSK5 or HPRT1 TaqMan probes (Thermo Fisher, Waltham, MA, USA) using TaqMan Fast Advanced Master Mix (Thermo Fisher, Waltham, MA, USA). The PCRs s were amplified in a QuantStudio 6 Flex Real-Time PCR for 40 cycles. After incubation at 95 °C for 20 s to activate Taq polymerase, the samples were amplified for 40 cycles with a 1 s denaturation step at 95 °C and a 20 s annealing/elongation step at 60 °C. The fold change in PCSK5 mRNA levels was calculated with the comparative Ct method and normalized to HPRT1.

### Statistical analysis

All data are presented as the means ± SEMs from at least three independent experiments unless otherwise indicated. The data were compared using Student’s t test or stated otherwise in the legend with GraphPad Prism 8.0 software (GraphPad Software Inc., San Diego, CA, USA). *P* < 0.05 was considered to be significant. (*P* values are reported for each experiment.)

## Supplementary information

Supplemental material Brodeur et al.

Figure S1

Figure S2

Figure S3

Figure S4

Figure S5

Figure S6

Figure S7

Supplemental material Tables

Uncropped Western blots Figure 1E

Uncropped Western blots Figure 8 and S7

Uncropped Western blots Figure S3

aj-checklist
