# Supplementary material for: Apoptotic exosome-like vesicles transfer specific and functional mRNAs to endothelial cells by phosphatidylserine-dependent macropinocytosis
Source: Cell Death Dis. 2023 Jul 20;14(7):449. doi: 10.1038/s41419-023-05991-x (PMC10359336; doi:10.1038/s41419-023-05991-x)
Supplement: Supplementary file 1 — Supplemental material Brodeur et al. [file 41419_2023_5991_MOESM1_ESM.docx]

Supplementary Materials for

**Apoptotic exosome-like vesicles transfer specific and functional mRNAs to endothelial cells by phosphatidylserine-dependent macropinocytosis**

Alexandre Brodeur^1,2,3,#^, Francis Migneault^1,2,#^, Maude Lanoie^1,2,3^, Déborah Beillevaire^1,2,3^, Julie Turgeon^1,2^, Annie Karakeussian Rimbaud^1^, Nicolas Thibodeau^1^,Éric Boilard^2,4^, Mélanie Dieudé^1,2,5,6^, and Marie-Josée Hébert^1,2,3^.

^1^Centre de recherche, Centre Hospitalier de l'Université de Montréal (CRCHUM) and Université de Montréal, Montréal, QC, Canada.

^2^Canadian Donation and Transplantation Research Program (CDTRP), Canada.

^3^Département de Médecine, Université de Montréal, Montréal, QC, Canada.

^4^Centre de Recherche, Centre Hospitalier Universitaire (CHU) de Québec-Université Laval, Département de Microbiologie et Immunologie Québec, QC, Canada.

^5^Département de Microbiologie, Infectiologie et Immunologie, Faculté de Médecine, Université de Montréal, Montréal, QC, Canada.

^6^Héma-Québec, Québec, QC, Canada.

# Both authors contributed equally to this work.

***Running title:*** **Apoptotic exosome-like vesicles transfer functional mRNA**

**Corresponding author**

*Dr. Marie-Josée Hébert, marie-josee.hebert@umontreal.ca

CRCHUM, 900 St. Denis Street, Pavilion R, Room R12.412, Montreal, QC, Canada H2X 0A9. Telephone: (514) 890-8000 Extension 28479, Fax: (514) 412-7944.

**DISCLOSURE**

The authors of this manuscript have no conflicts of interest to disclose.

**Supplementary Materials and Methods**

*Murine primary renal endothelial cells (MPREC) cell culture*. MPREC from C57BL/6 mice were purchased from CellBiologics (C57-6014), grown in Complete Endothelial Cell Medium (CellBiologics, M1168) and used between passages 6 and 8. Cells were exposed to experimental conditions or vehicle when they reached 80-90% confluence.

*Aortic murine cell isolation*. Murine ECs (mEC) were isolated from the aorta of C57BL/6 (wild-type; WT) grown in Dulbecco's modified eagle medium (DMEM) low‐glucose culture media supplemented with endothelial cell growth supplements (ECGS; Alfa Aesar, Haverhill, MA), 10% fetal bovine serum (FBS; Invitrogen, Carlsbad, CA), 10% newborn calf serum (Invitrogen), heparin (12.6 U/mL, Sandoz, Holzkirchen, Germany), 1% penicillin‐streptomycin, and 1% amphotericin B. To generate conditioned media, cells were exposed to serum-free medium RPMI-1640 (Invitrogen) for 9 hours. Conditioned medium from mEC cultures was fractionated by sequential centrifugation as described for HUVEC-derived conditioned medium.

*WI38 human fibroblasts cell culture*. WI-38 human fibroblasts from normal embryonic lung tissue were purchased from the American Type Culture Collection (CCL-75), grown in fibroblast basal medium (Lonza, CC-3131) supplemented with 10% inactivated fetal bovine serum (Wisent, 090150; normal growth medium [N]) and used between passages 6 and 8. Cells were plated at a density of 20,000 cells/cm^2^ in 6-well plates and exposed to experimental conditions or vehicle when they reached 80-90% confluence.

**Table S1. List of the protein-coding transcripts expressed in ApoExo, ApoBodies and endothelial cells in normal (HUVEC_N) or serum-starved (HUVEC_SS) conditions.**

**Tables S2-4. Fold enrichment and p-value for each GO-term associated to biological processes, cellular components and molecular function.**

**Figure S1. Apoptotic exosome-like vesicles are different from classical exosomes and apoptotic bodies.** Small-particle flow cytometry gating strategy to analyze the expression of phosphatidylserine and proteasome on CellTrace + EVs in conditioned media from endothelial cells. Size calibration of the flow cytometer by using silica particles of known dimensions (100 nm, 500 nm, 1 μm and 2 µm in diameter). Control experiments with small particle flow cytometry to validate the particle size, nature and membrane moiety of extracellular vesicles by using respectively silica particles, ultracentrifugation depletion, Ca2+ ion chelation and detergent treatment. n ≥ 3 for each condition.

**Figure S2.** **Apoptotic exosome-like vesicles internalization is mediated by phosphatidylserine-dependent macropinocytosis.** (**A**) Quantification of ApoBodies and ExoN uptake by serum-starved endothelial cells treated at 37 °C or 4 °C for 1 h using flow cytometry. n ≥ 3 for each condition. (**B**) Time-dependent uptake of protein-labeled ApoExo by murine primary renal endothelial cells (mPRECs) and human fibroblasts (WI38) treated at 37°C or 4 °C for 1 to 4 h quantified by flow cytometry. n ≥ 3 for each condition. (**C**) Macropinocytosis inhibition suppressed ApoExo uptake by mPRECs and WI38. Quantification of ApoExo uptake by flow cytometry in mPRECs and WI38 pre-treated for 30 min with EIPA 50 µM or its vehicle (DMSO; Ctrl) followed by a treatment of 1 h. n ≥ 3 for each condition. Flow cytometry experiments expressed as median fluorescence intensity (MFI) (30,000 events/sample) ± SEM. P values were obtained by one-way ANOVA and Bonferroni's *post hoc* test. (* P < 0.05, ** P < 0.01, and *** P < 0.001).

**Figure S3. Representative immunoblot of CAV1 knock-down and β-actin expression.** Serum-starved endothelial cells were transfected with Ctrl or CAV1 siRNA 90 nM. CAV1 expression was normalized with β-actin expression and represented as arbitrary units ± SEM. n = 3 for each condition. P value was obtained by unpaired t-test. (*** P < 0.001).

**Figure S4. Macropinocytosis acts as the central pathway for ApoExo uptake.**(**A**) Quantification by flow cytometry of ApoExo, apoptotic bodies and normal exosomes uptake in serum-starved endothelial cells pre-treated for 30 min with EIPA 50 µM or its vehicle (DMSO; Ctrl) followed by a treatment of 1 h. n ≥ 3 for each condition. (**B**) Quantification by flow cytometry of the uptake of ApoExo pre-treated for 1 h with vehicle (water; Ctrl) or annexin V 10 µg/mL then incubated with serum-starved endothelial cells pre-treated for 30 min with vehicle (DMSO; Ctrl) or EIPA 50 µM followed by a treatment of 1 h. n ≥ 3 for each condition. Flow cytometry experiments expressed as median fluorescence intensity (MFI) (30,000 events/sample) ± SEM. P values obtained by unpaired t-test (* P < 0.05, ** P < 0.01, *** P < 0.001 and **** P < 0.0001).

**Figure S5. ApoExo transfer encapsulated RNA to endothelial cells**. Quantification by flow cytometry of the uptake of ApoExo pre-treated for 30 min with RNAse A (5 μg/mL) then incubated with serum-starved endothelial cells for 1 h. n = 3 for each condition. Flow cytometry experiments expressed as median fluorescence intensity (MFI) (30,000 events/sample) ± SEM. P values were obtained by unpaired t-test. (* P < 0.05, ** P < 0.01).

**Figure S6. ApoExo express a specific profile of mRNAs.** (**A**) Principal component analysis (PCA) using protein-coding mRNAs in extracellular vesicles and cells. (**B**) Gene ontology analysis of cellular component and molecular function for mRNA enriched in ApoExo.

**Figure S7. ApoExo transfer functional PCSK5 mRNA to endothelial cells increasing PCSK5 protein levels.** (**A**) Serum-starved endothelial cells were treated with ApoExo or conditioned media depleted of extracellular vesicles for 24 h. Control (Ctrl) represents cells treated with RPMI serum-free medium. n = 3 for each condition. (**B**) Endothelial cells were transfected with Ctrl or PCSK5 siRNA 90 nM. Forty-eight hours post-transfection, cells were exposed to RPMI serum-free medium for 4 hours to produce PCSK5-depleted ApoExo. Expression of PCSK5 mRNA was measured by quantitative RT-PCR and presented as relative expression of PCSK5 mRNA compared to cells transfected with control siRNA (siCtrl) ± SEM after normalization with HPRT1.; n ≥ 3 for each condition. (**C**) Endogenous PCSK5 protein expression is not modulated by the macropinocytosis inhibitor EIPA. Endothelial cells were exposed to the vehicle (DMSO) or EIPA 30 µM (EIPA) for 24 h. PCSK5 expression was quantified by densitometry and expressed as arbitrary units ± SEM; n ≥ 3. Representative immunoblots cropped from the same gel are presented. (**D**) PCSK5 protein is not expressed in ApoExo. Western Blots showing PCSK5 and 20S proteasome in ApoExo and cell extract. Molecular weights expressed in kDa. Representative blots are depicted. n ≥ 3 for each condition. P values obtained by unpaired t-test (* P < 0.05, ** P < 0.01, and *** P < 0.001).
