# Supplementary figures and images for: Apoptotic exosome-like vesicles transfer specific and functional mRNAs to endothelial cells by phosphatidylserine-dependent macropinocytosis
Source: Cell Death Dis. 2023 Jul 20;14(7):449. doi: 10.1038/s41419-023-05991-x (PMC10359336; doi:10.1038/s41419-023-05991-x)

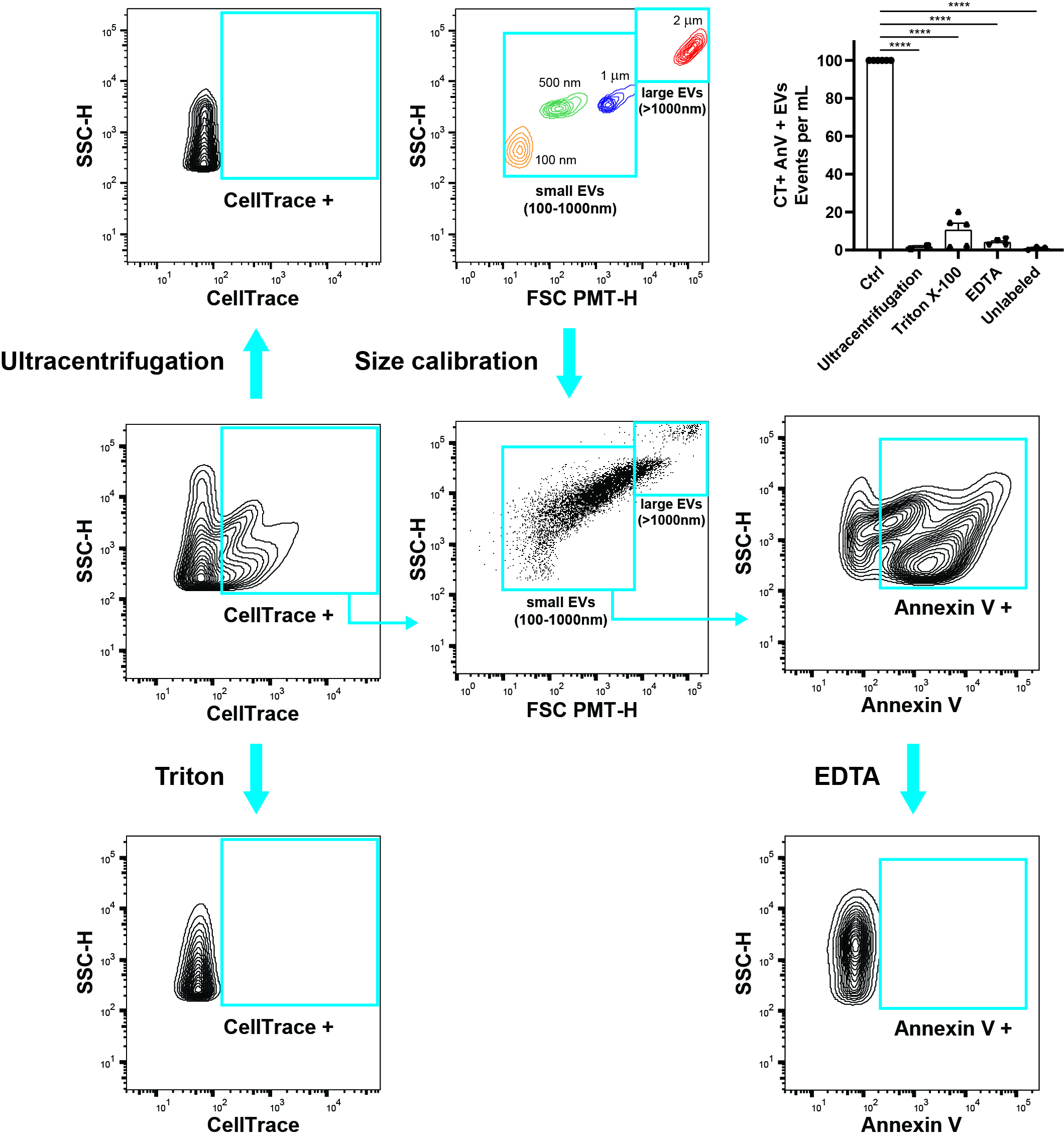

Supplement: Supplementary file 2 — Figure S1 [file 41419_2023_5991_MOESM2_ESM.tif]

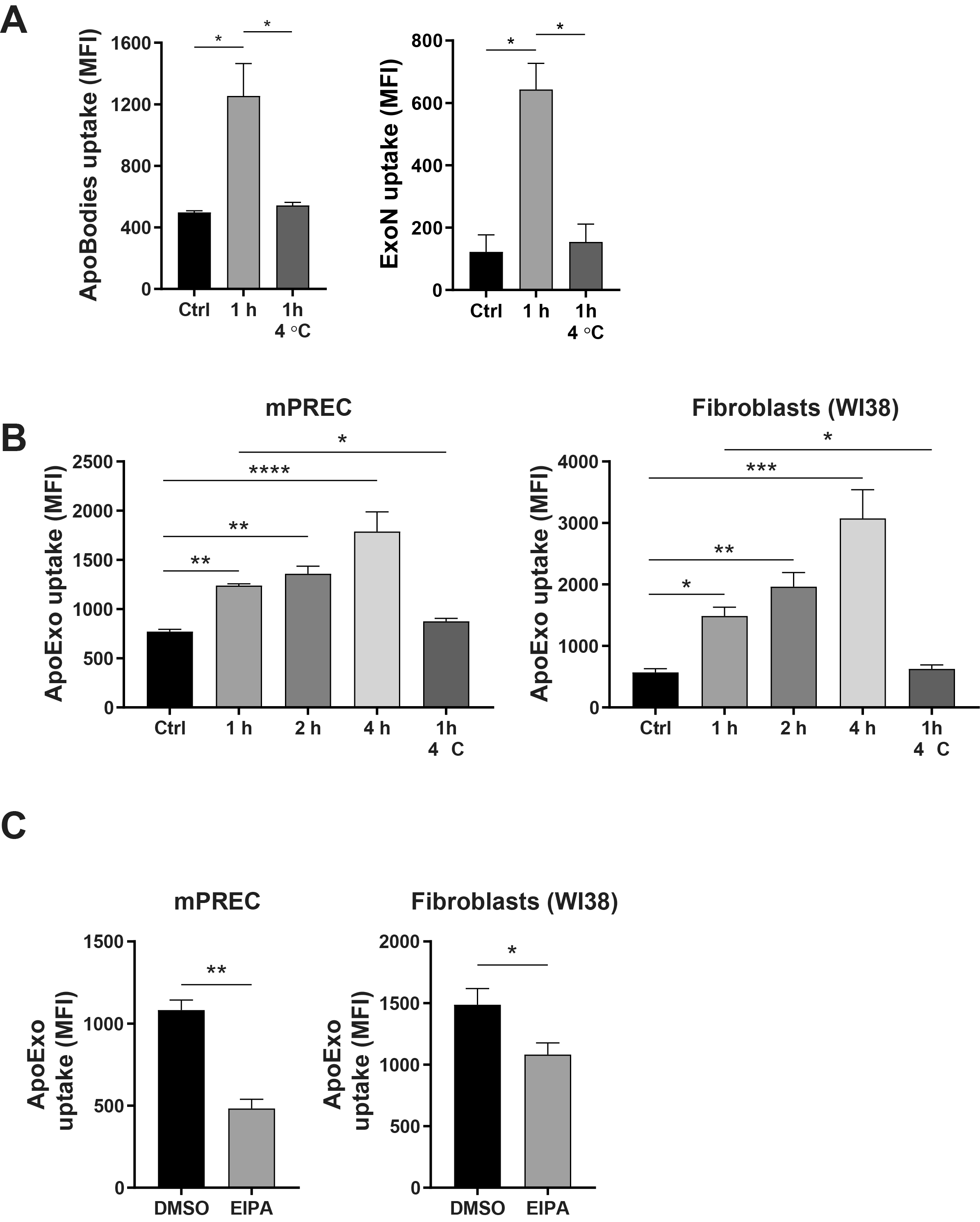

Supplement: Supplementary file 3 — Figure S2 [file 41419_2023_5991_MOESM3_ESM.tif]

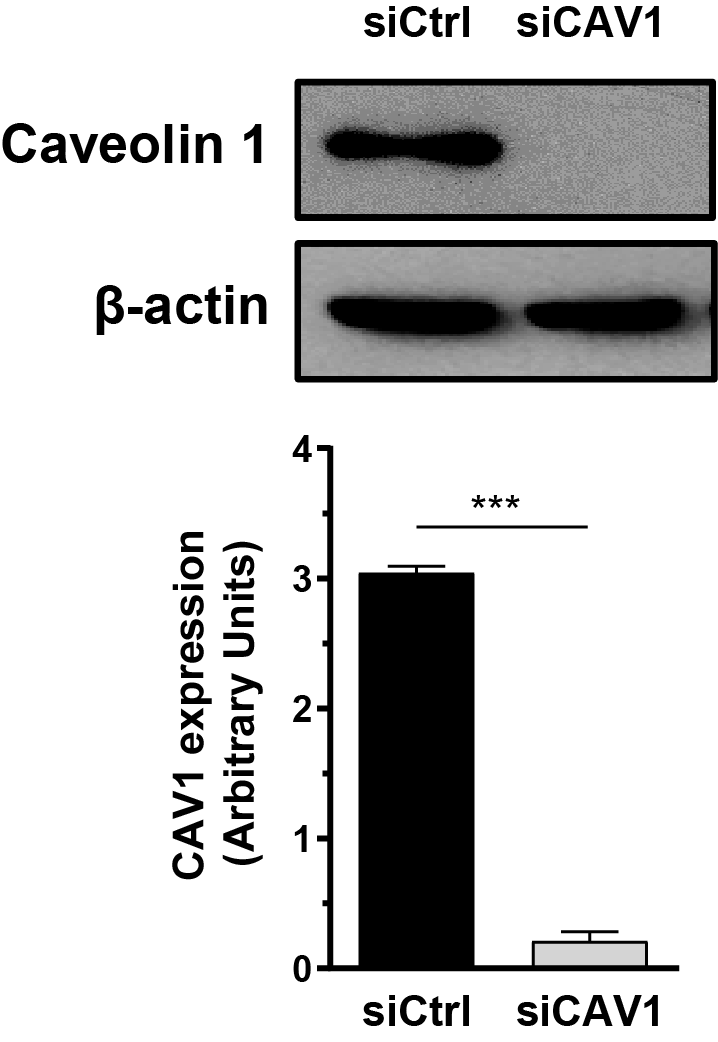

Supplement: Supplementary file 4 — Figure S3 [file 41419_2023_5991_MOESM4_ESM.tif]

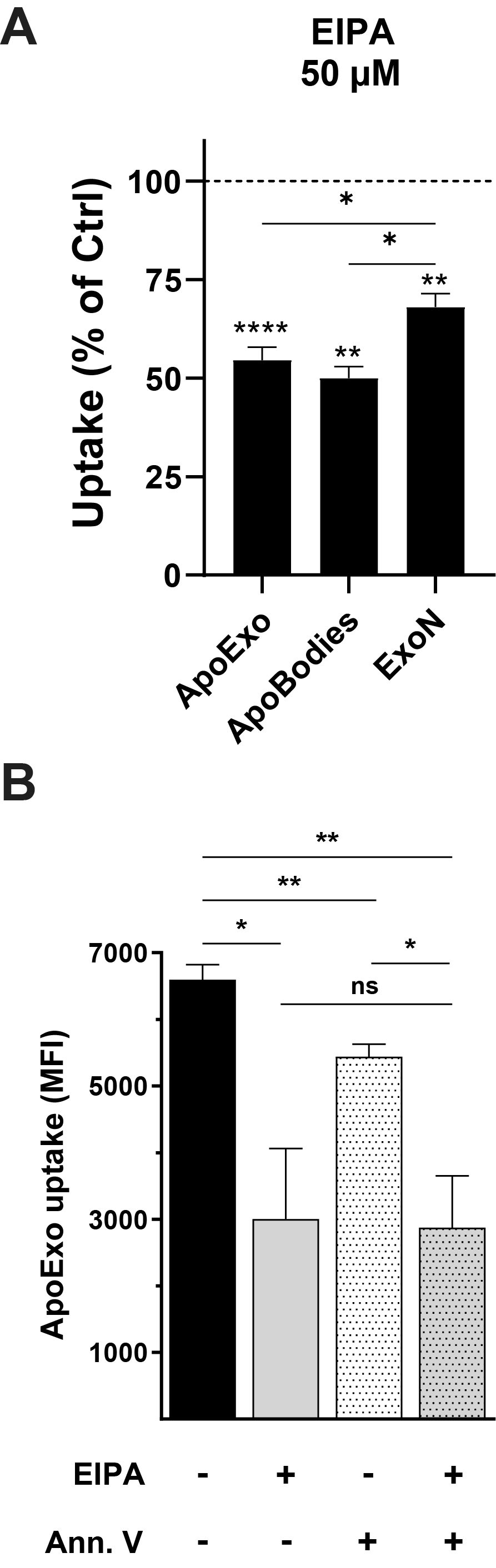

Supplement: Supplementary file 5 — Figure S4 [file 41419_2023_5991_MOESM5_ESM.tif]

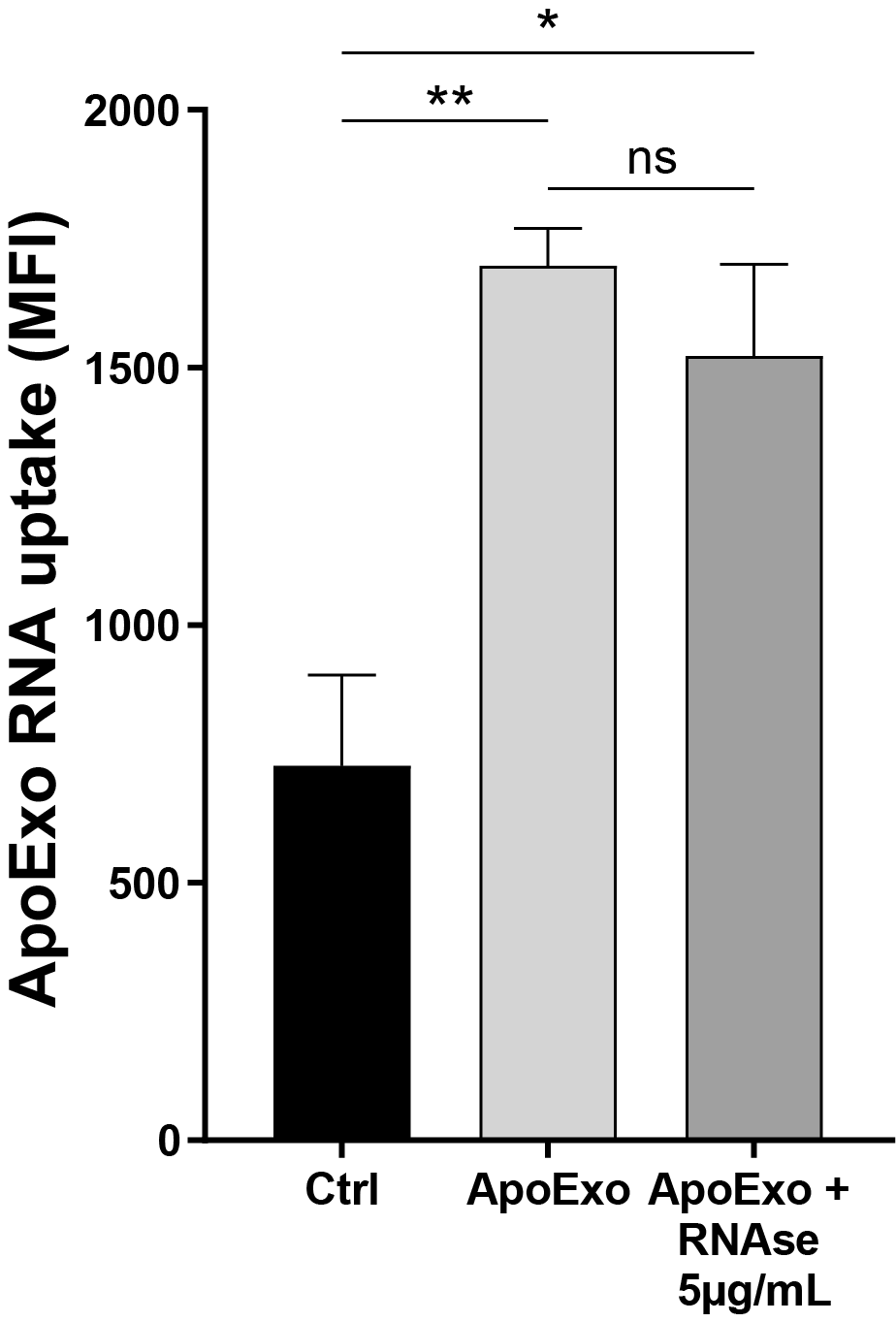

Supplement: Supplementary file 6 — Figure S5 [file 41419_2023_5991_MOESM6_ESM.tif]

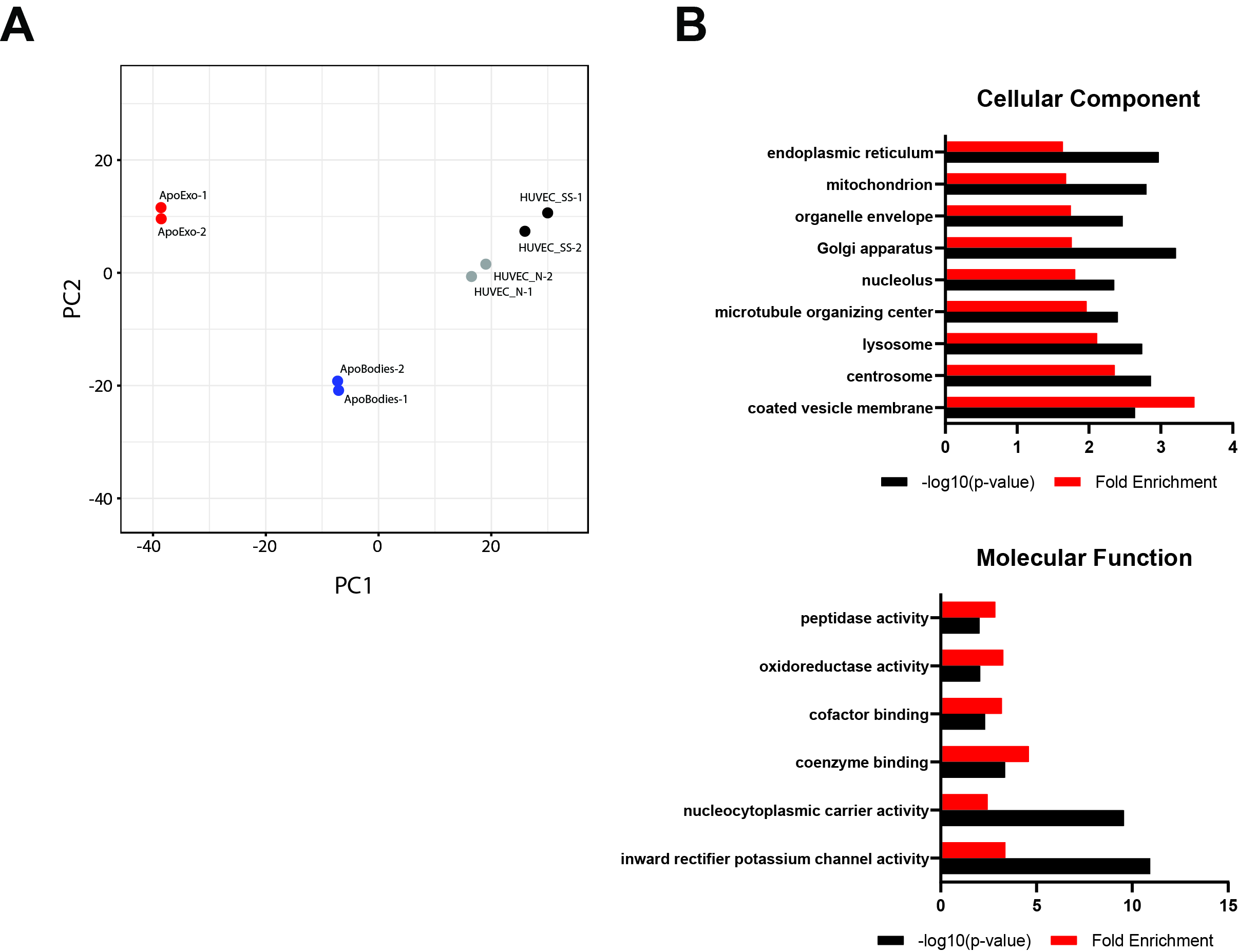

Supplement: Supplementary file 7 — Figure S6 [file 41419_2023_5991_MOESM7_ESM.tif]

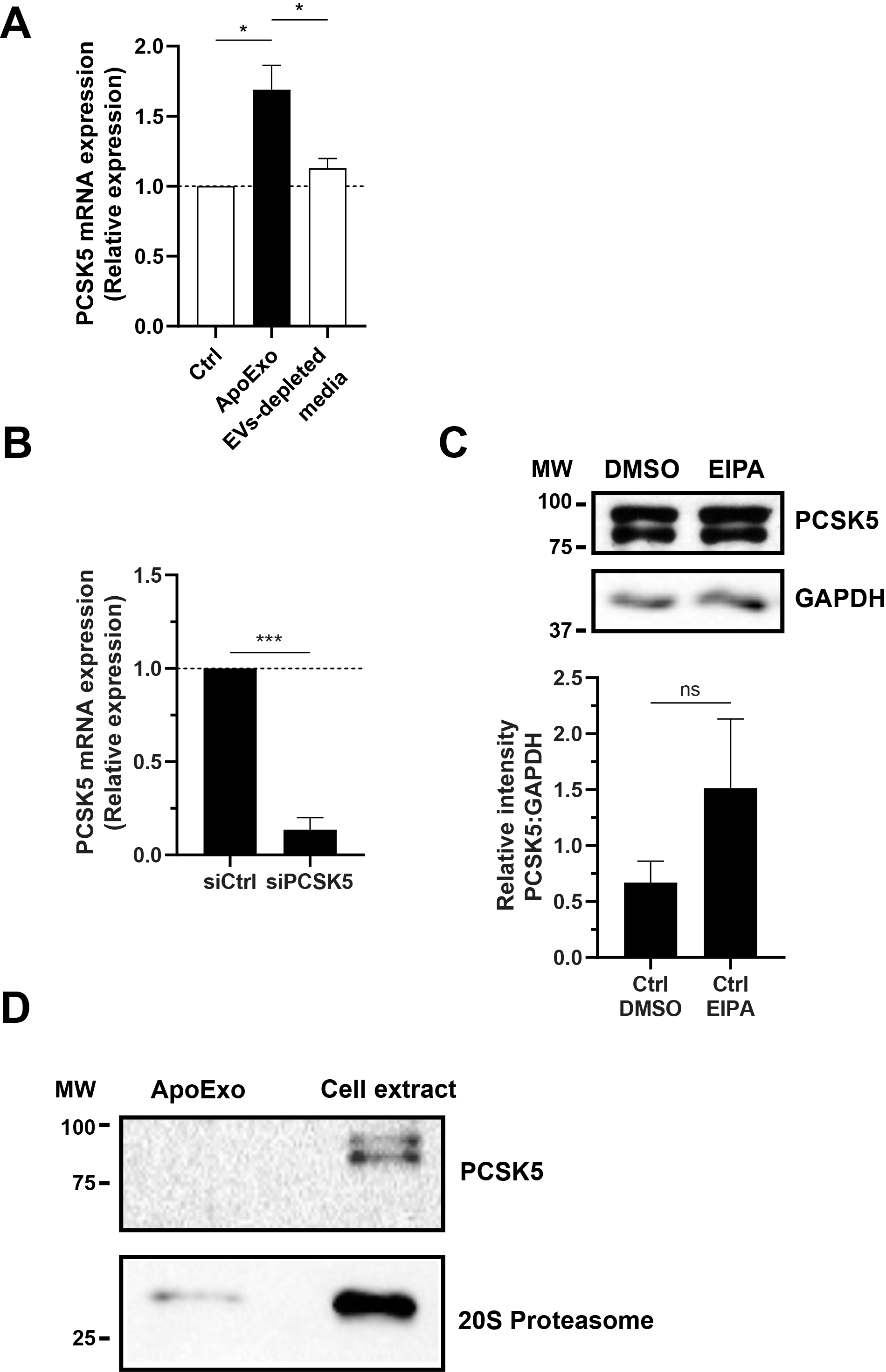

Supplement: Supplementary file 8 — Figure S7 [file 41419_2023_5991_MOESM8_ESM.tif]

# Original Immunoblots

Figure 1E

**CD63**

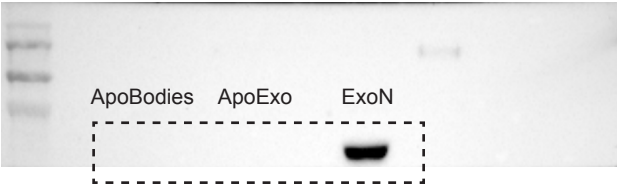

**LG3**

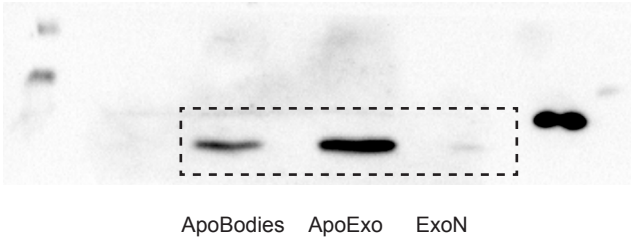

**CD82**

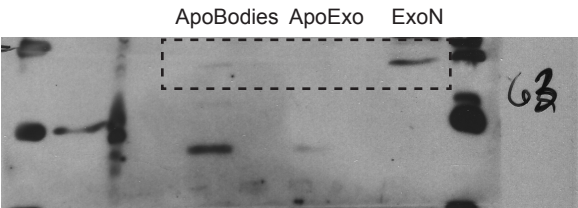

**Tubulin**

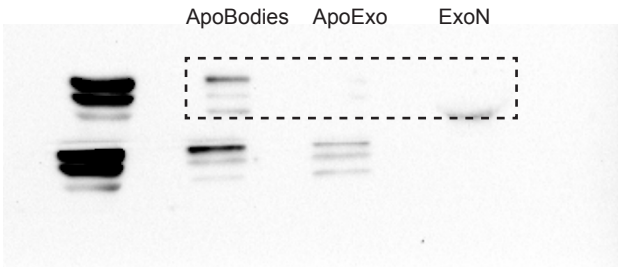

**LAMP2**

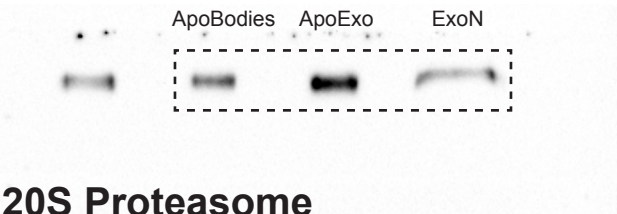

**20S Proteasome**

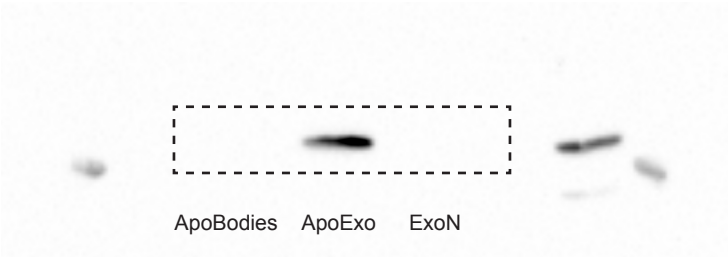

**GM130**

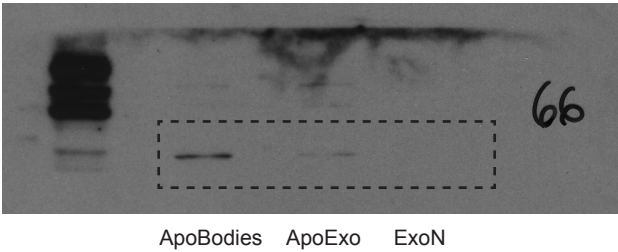

Supplement: Supplementary file 10 — Uncropped Western blots Figure 1E [file 41419_2023_5991_MOESM10_ESM.pdf]

# Original Immunoblots

Figure 8 and S7

PCSK5

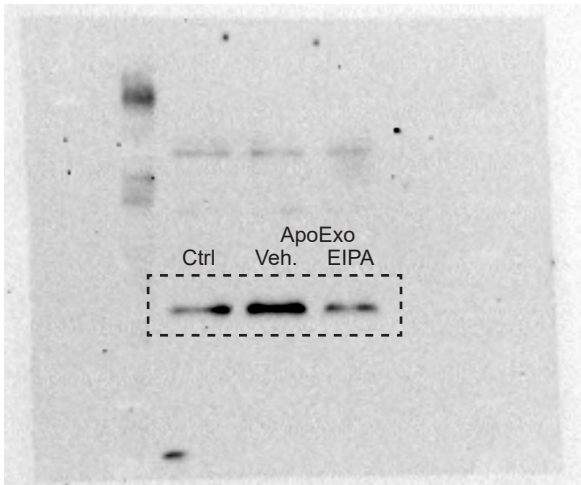

GAPDH

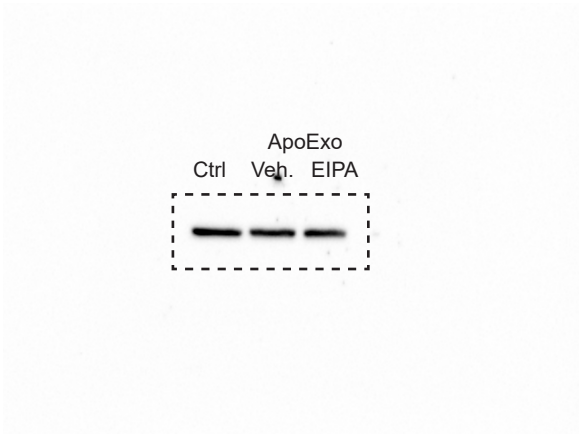

PCSK5

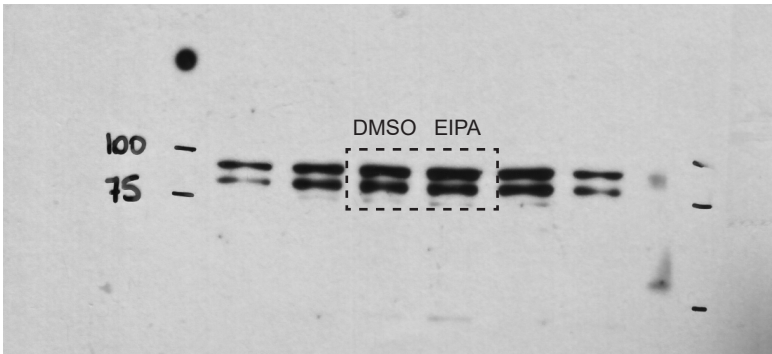

PCSK5

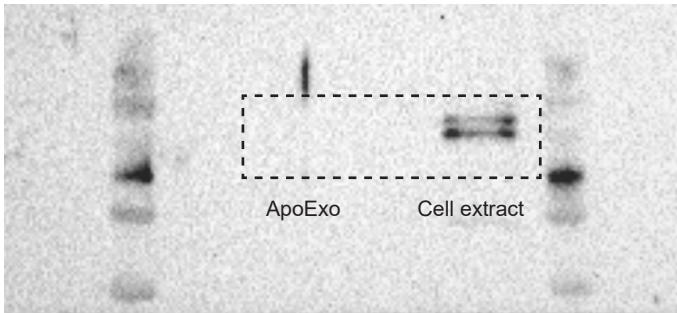

GAPDH

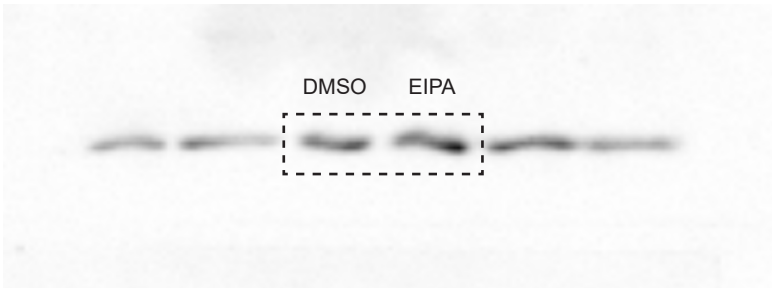

20S Proteasome

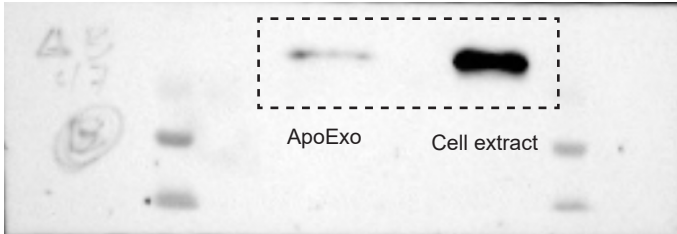

Supplement: Supplementary file 11 — Uncropped Western blots Figure 8 and S7 [file 41419_2023_5991_MOESM11_ESM.pdf]

# Original Immunoblots

Figure S3

## Caveolin 1

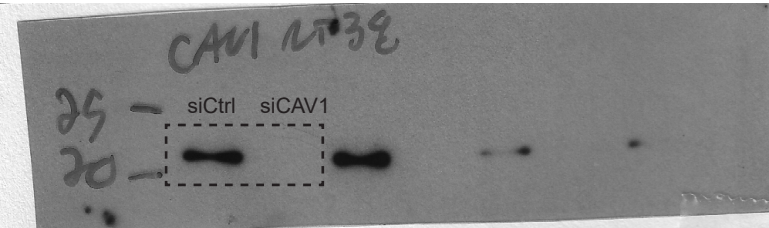

## B-actin

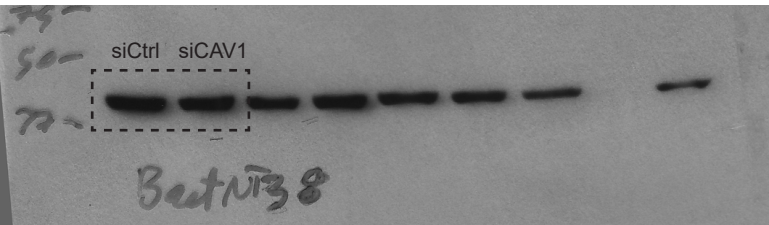

Supplement: Supplementary file 12 — Uncropped Western blots Figure S3 [file 41419_2023_5991_MOESM12_ESM.pdf]
